# Dressed Up to the Nines: The Interplay of Phytohormones Signaling and Redox Metabolism During Plant Response to Drought

**DOI:** 10.3390/plants14020208

**Published:** 2025-01-13

**Authors:** Douglas Jardim-Messeder, Ygor de Souza-Vieira, Gilberto Sachetto-Martins

**Affiliations:** 1 Departamento de Genética, Instituto de Biologia, Universidade Federal do Rio de Janeiro, Rio de Janeiro 21941-902, Brazil; ygor.vieira3126@gmail.com; 2 Programa de Biologia Molecular e Biotecnologia, Instituto de Bioquímica Médica Leopoldo de Meis, Universidade Federal do Rio de Janeiro, Rio de Janeiro 21941-902, Brazil

**Keywords:** auxins, brassinosteroids, cytokinins, gibberellins, abscisic acid, ethylene, jasmonic acid, salicylic acid, strigolactones, reactive oxygen species

## Abstract

Plants must effectively respond to various environmental stimuli to achieve optimal growth. This is especially relevant in the context of climate change, where drought emerges as a major factor globally impacting crops and limiting overall yield potential. Throughout evolution, plants have developed adaptative strategies for environmental stimuli, with plant hormones and reactive oxygen species (ROS) playing essential roles in their development. Hormonal signaling and the maintenance of ROS homeostasis are interconnected, playing indispensable roles in growth, development, and stress responses and orchestrating diverse molecular responses during environmental adversities. Nine principal classes of phytohormones have been categorized: auxins, brassinosteroids, cytokinins, and gibberellins primarily oversee developmental growth regulation, while abscisic acid, ethylene, jasmonic acid, salicylic acid, and strigolactones are the main orchestrators of environmental stress responses. Coordination between phytohormones and transcriptional regulation is crucial for effective plant responses, especially in drought stress. Understanding the interplay of ROS and phytohormones is pivotal for elucidating the molecular mechanisms involved in plant stress responses. This review provides an overview of the intricate relationship between ROS, redox metabolism, and the nine different phytohormones signaling in plants, shedding light on potential strategies for enhancing drought tolerance for sustainable crop production.

## 1. Introduction

Plants, as sessile organisms, must respond effectively to environmental stimuli to achieve optimal development. Different environmental factors have the potential to inflict stress on plants, limiting their growth. Abiotic stresses, manifested in different ways, are frequently associated with changes in climatic conditions, such as rain, temperature, solar irradiation, and soil quality. Changes in water availability and temperature, leading to hydric and heat stresses, have been related to climate change’s impact [1]. The integration of models on climate change and agricultural production considers that, soon, the abrupt impact of changes in environmental conditions will intensify, representing a greater risk to the productivity of crops. Given plants’ crucial role as a primary food source, this situation could have serious implications for food security in a rapidly growing global population. Moreover, plants are important fuel and fiber sources, contributing significantly to production diversity and ecological sustainability [2,3].

Through evolution, plants have developed adaptive strategies to respond to environmental challenges to ensure their reproductive success. These strategies include a series of morphological, anatomical, and metabolic adjustments to better cope with the environmental conditions. Among these strategies is the crucial importance of reactive oxygen species (ROS) as secondary messengers as well as the signaling mediated by phytohormones, which perform an essential function in plant development and reproduction [4,5]. The integration between hormonal signaling and the management of ROS homeostasis controls diverse types of signaling, especially those involved with stress responses [6].

Many of the signaling pathways in which ROS act as signaling molecules are orchestrated by phytohormones [7], which play an indispensable role in metabolism, development, coordination, stress response, and even in death [8]. Plants synthesize various phytohormones, such as abscisic acid (ABA), ethylene (ET), salicylic acid (SA), jasmonic acid (JA), gibberellins (GA), auxin (AUX), cytokinin (CK), brassinosteroids (BRs), and strigolactones (SL), to orchestrate and regulate various aspects of growth and development. These phytohormones play a fundamental role in a myriad of dynamic yet rigorously regulated molecular responses throughout the plant life cycle. Responding to environmental adversities, these phytohormones play a crucial regulatory role in gene expression machinery, preparing the plant to withstand unforeseen circumstances [9,10,11].

Among the different types of abiotic stress, drought emerges as the main factor that harms crops globally and directly impacts harvests, ultimately restricting total yield potential [12]. The primary mechanisms controlling drought response involve stomatal closure, modulation of root growth and architecture, and the upregulation of anti-stress proteins [13]. As additional strategies that contribute to mitigating the adverse effects of water stress, cells reduce their hydric potential (ψ), leading to the accumulation of specialized solutes. All these multivariate responses are mainly orchestrated by various hormonal regulations, which support cellular plasticity and help plants develop an effective coping strategy against drought [14]. During water shortage, phytohormones regulate plant growth and development, resulting in increased antioxidant enzyme production, secondary metabolites, and heat-shock proteins [15]. Several studies have indicated that communication between ROS and phytohormones signaling is essential to initiate and modulates the response of tolerance of different stresses, including drought. Therefore, precise coordination in the interaction with phytohormones is essential for the appropriate response in plants [7].

To develop comprehensive concepts and strategies for safeguarding plants against the deleterious effects of abiotic stress and meeting the future demands for plant products, an in-depth exploration of molecular-level mechanisms governing plant stress responses is imperative. This review focuses on elucidating the intricate roles played by ROS, redox metabolism, and signaling mediated by the different phytohormones classes—abscisic acid, ethylene, salicylic acid, jasmonic acid, gibberellins, auxin, cytokinin, brassinosteroids, and strigolactones—in the context of drought stress. The objective is to provide a comprehensive overview of the existing knowledge about how these signaling cascades and redox metabolic processes synergize, leading to robust responses to water stress in plants.

## 2. Abscisic Acid

ABA is a phytohormone with a central role in the response to water and osmotic stress. ABA orchestrates a diverse range of physiological processes, from seed germination to stomatal regulation. Simultaneously, it controls plant growth and development by regulating the synthesis of protective metabolites. These metabolites enable plants to combat the detrimental effects of stressful environments, which is why ABA is often referred to as the “universal stress hormone”.

### 2.1. Abscisic Acid Metabolism

In the ABA biosynthesis pathway, the enzyme 9-cis-epoxycarotenoid dioxygenase (NCED; EC 1.13.11.51) acts as a bottleneck in the conversion of 9-neoxanthins to xanthin. This enzymatic process influences ABA levels during drought response. The subsequent enzymatic cleavage of ABA into 80-OH-ABA, catalyzed by ABA 8-hydroxylase (CYP707A; EC 1.14.14.137), represents an inactivation mechanism, resulting in dehydrophaseic acid (DPA) and phaseic acid (PA) as the main degradation products [16]. ABA, primarily produced in leaf vascular tissues in response to water deficit stress, is transported to guard cells, where it triggers stomatal closure. It is believed that ABA is transported via passive diffusion from a low- to a high-pH environment due to its nature as a weak acid, without the need for ABA transporters [10]. However, ABA can also be transported by specific ABA transporters, particularly members of the ABC subfamily G [17] and the NPF (Nitrate transporter 1/peptide transporter family) [18].

### 2.2. Abscisic Acid Signaling

**Figure 1:**
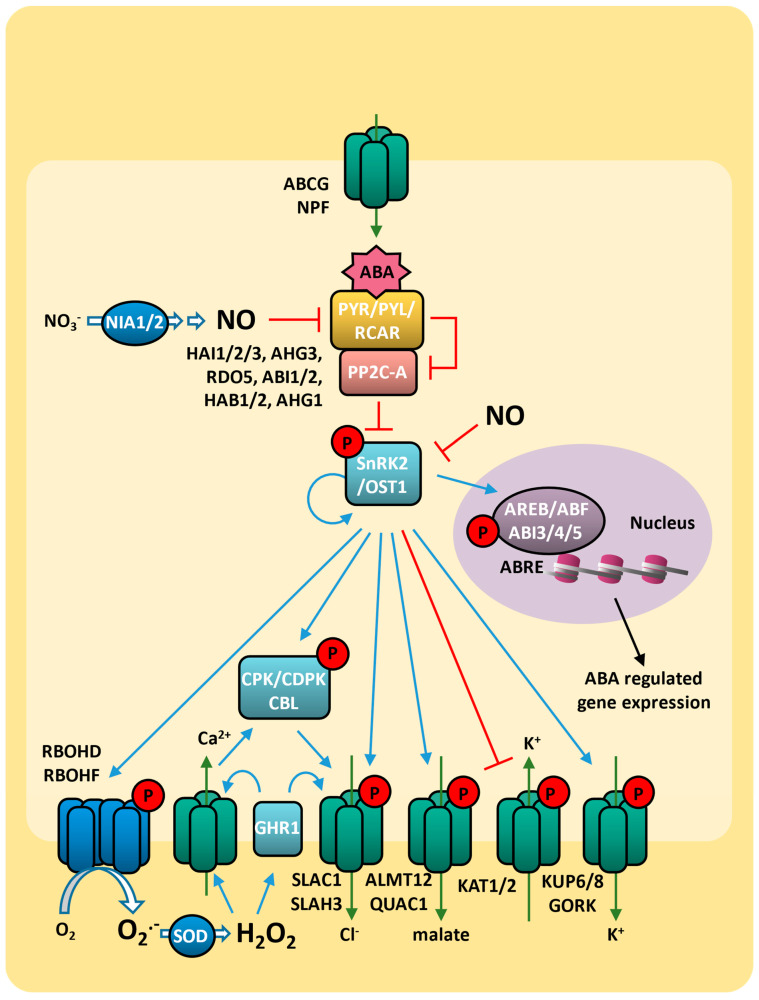
ABA signaling. ABA signaling integrates multiple molecular components to regulate both stomatal responses and gene expression under stress conditions. ABA is transported across cellular membranes by ABCG and NPF transporters. ABA binding to its receptor, PYR/PYL/RCAR, leads to the deactivation of the PP2C-A phosphatases such as HAI1, HAI2, HAI3, AHG3, RDO5, ABI1, ABI2, HAB1, HAB2, and AHG1. This inhibition activates SnRK2 kinases, which phosphorylate transcription factors including AREB, ABF, ABI3, ABI4, and ABI5, promoting the expression of ABA-responsive genes. In guard cells, SnRK2 kinases also phosphorylate ion channels SLAC1, SLAH3, ALMT12, QUAC1, KAT1, KAT2, KUP6, KUP8, and GORK, regulating ion flux. This mechanism drives osmotic water efflux, reducing turgor and resulting in stomatal closure. Additionally, SnRK2 kinases activate NADPH oxidases RBOHD and RBOHF, which increase ROS production. ROS amplify stress signaling by activating plasma membrane receptor kinase GHR1 and redox-sensitive calcium channels, leading to calcium influx and the activation of calcium-dependent protein kinases (CPKs), including CDPKs and CBLs. Both GHR1 and CPKs further phosphorylate SLAC1 and SLAH3, supporting ion flux changes. Furthermore, nitrate reductases NIA1 and NIA2 influence stomatal response through nitrogen metabolism, generating NO, which is known to inhibit the PYR/PYL receptor, fine-tuning the ABA response under various stress conditions. The hormonal receptor is indicated in yellow, while activators of the signaling are shown in blue and inhibitors in salmon. Membrane channels/transporters are represented in green, transcription factors in purple, and enzymes involved in ROS metabolism in dark blue. Black lines with arrows indicate activation, blue lines with arrows indicate activation of phytohormone signaling, red lines with bars represent inhibition, and green lines with arrows indicate transmembrane transport.

Under basal conditions, plants produce ABA at low concentrations [19], and the kinase activity of SnRK2 (sucrose nonfermenting 1 (SNF1)-related protein kinase 2) is inhibited by the phosphatase PP2C. In arabidopsis (*Arabidopsis thaliana*), PP2Cs from clade “A”, such as HAI1 (HIGHLY ABA-INDUCED 1), HAI2, HAI3, AHG1 (ABA-HYPERSENSITIVE GERMINATION 1), AHG1, RDO5 (REDUCED DORMANCY 5), ABI1 (ABA-INSENSITIVE 1), ABI2, HAB1 (HYPERSENSITIVE TO ABA1), and HAB2, have been identified as important regulators of ABA signaling [20,21]. The increase in ABA concentration triggers ABA signaling, which involves the recognition of ABA by PYR/PYL/RCARs receptors, leading to the inactivation of PP2C phosphatase activity (Figure 1) [22,23].

The PYR/PYL/RCAR proteins are encoded by various genes, acting as essential receptors for the ABA response. The autoactivation of SnRK2/OST1 is triggered when the protein dissociates from the PP2C phosphatases. In arabidopsis, the isoforms SnRK2.2, SnRK2.3, and SnRK2.6 are primarily involved in the ABA response and function by phosphorylating different targets [21,24].

The ABA signaling cascade involves several transcription factor branches, and the cis-acting element known as the ABA-responsive element (ABRE) is present in the promoters of genes induced by drought [25]. These ABREs function as binding sites for leucine zipper transcription factors, such as ABRE binding protein/ABRE binding factors (AREBs/ABFs) and ABI3 (ABA-INSENSITIVE 3), ABI4, and ABI5 [25]. During ABA signaling, ABA-dependent SnRK2 protein kinases phosphorylate and directly activate the AREB/ABF and ABI transcription factors [24,26] (Figure 1).

ABA-responsive elements contribute significantly to the complexities of ABA signaling [27]. SnRK2 activation triggers the induction of molecular and physiological responses to ABA, such as the modulation of photosynthetic activity, root growth, and germination [21]. In addition to these direct effects, ABA exerts regulatory control over a series of genes associated with drought responses, substantially contributing to drought tolerance development in plants [15,28]. This finding, coupled with the identification of numerous classic ABA-insensitive mutations located in transcriptional regulators, strongly suggests a central role in gene regulation, conferring resistance to abiotic stress [29].

### 2.3. Abscisic Acid and ROS on Drought Response

During drought response, ABA also plays an important role in regulating ROS production, as hydrogen peroxide is generated mainly by the interaction and phosphorylation of NADPH oxidases (known as respiratory burst oxidase homologs, (RBOHD and RBOHF)) via SnRK2 [30,31] (Figure 1). Furthermore, SnRK2-dependent hydrogen peroxide production can act as a signal for further release of active SnRK2 through PP2C inactivation [32]. Consequently, ABA and ROS act in a positive feedback cycle, resulting in elevated levels of ROS/ABA that regulate gene expression and facilitate cellular responses to stress [33].

The ABA-mediated ROS production controls stomatal opening by activating Ca^2+^ channels in guard cells, triggering an increase in the concentration of cytoplasmic Ca^2+^ ([Ca^2+^]cyt) that then activates SLAC1 (slow anion channel 1) via CPK/CDPK (calcium-dependent protein kinase) or CBL (calcinuerin B-like) [17]. The ABA-induced hydrogen peroxide activates guard cells’ GHR1 membrane receptor protein kinase (GUARD CELL HYDROGEN PEROXIDE-RESISTANT 1), which interacts and activates SLAC1 [34]. Both CPK and GHR1 are also able to activate SLAH3 (SLAC1 homolog 3), promoting the Cl^−^ efflux [35]. This effect is accompanied by the regulation of other membrane ion transporters by SnRK2, such as the activation of outward shaker K^+^ channels GORK, KUP6, and KUP8 and the inhibition of both inward K^+^ channels KAT1/KAT2 and the aluminum-activated malate transporter ALMT12/QUAC1 channel (quick anion channel 1) [17] (Figure 1). Anion and K^+^ effluxes cause membrane depolarization and reduction in guard cell turgor, ultimately leading to reduced stomatal aperture size and minimized water loss through transpiration [33,36]. This mechanism is directly related to increased hydrogen peroxide levels: the double mutant *atrbohd*/*atrbohf*, which generates lower ABA-induced ROS, showed impaired ABA-induced Ca^2+^ channels and stomatal closure [30]. This phenotype is rescued by exogenous hydrogen peroxide, indicating that ROS serves as a secondary messenger in ABA signaling of stomatal response [37]. ABA-induced hydrogen peroxide also activates NIA1 and NIA2 (nitrate reductase 1 and 2; EC 1.7.1.1), leading to NO (nitric oxide) synthesis, which is also critical for ABA-induced stomatal closure [38]. NO negatively regulates abscisic acid signaling in guard cells by inhibition of ABA receptor PYR/PYL/RCAR [39] and SnRK2 [40] (Figure 1). NO also reduces ABA content by the induction of the expression of ABA catabolic gene *CYP707A* and the inhibition of ABA biosynthesis-related gene NCED1 [41]. Indeed, enhanced sensitivity to ABA during development and in responses to stress is observed in the NO-deficient arabidopsis triple mutant *nia1 nia2 noa1-2* [42]. ROS signaling also plays a significant role in water stress tolerance through ABA-induced transcription factors. In arabidopsis, RRTF1 (redox-responsive transcription factor 1), belonging to the ERF/AP2 (ethylene response factor 1; APETALA 2) family, is a key component of the central redox signaling network. This network is activated by both ABA and ROS in response to various stresses, including drought. Elevated levels of RRTF1 lead to additional ROS accumulation, playing an important role in initiating subsequent acclimation responses [43,44].

During water stress, ABA also regulates root tissue patterning. In the endoderm, ABA signaling promotes xylem differentiation by inducing the expression of microRNAs miR165 and miR166, both essential regulators of vascular development [45]. In addition, ABA acts directly in xylem cells, activating the expression of *VND* (*vascular-related NAC domain*) transcription factors that promote xylem differentiation [46].

## 3. Ethylene

Ethylene is a gaseous phytohormone, which governs various physiological processes and responses to environmental stimuli, regulating leaf abscission, flowering, fruit ripening, senescence, seed germination, cell division, tissue differentiation, sex determination, adventitious root growth, starch accumulation epinasty, stomatal closure, etc. [47,48,49,50,51,52,53,54,55]. Moreover, it acts as a triple-response growth regulator, influencing shoot elongation, stem thickening, and horizontal growth habit [47], and it serves as a stress hormone, mediating plant reactions to biotic and abiotic stresses like pathogen attacks, salinity, drought, hypoxia, cold, and heat [56,57,58,59,60,61].

### 3.1. Ethylene Metabolism

The ethylene biosynthesis pathway starts with the amino acid methionine, which is converted to S-adenosyl-L-methionine (SAM) by S-adenosylmethionine synthetase (SAMS; EC 2.5.1.6) in the Yang cycle [62,63]. SAM is then converted into 1-aminocyclopropane-1-carboxylic acid (ACC) and 5-methylthioadenosine (MTA) by ACC synthase (ACS, EC 4.4.1.14) [63]. While MTA is recycled through the Yang cycle reactions back to methionine [64], ACC is oxygenated by ACC oxidase (ACO, EC 1.14.17.4; also named ethylene forming enzyme—EFE) to produce ethylene [65]. Ethylene biosynthesis is regulated mainly by the transcriptional and post-translational control of ACS and ACO steps by different internal and external stimuli [66].

### 3.2. Ethylene Signaling

**Figure 2:**
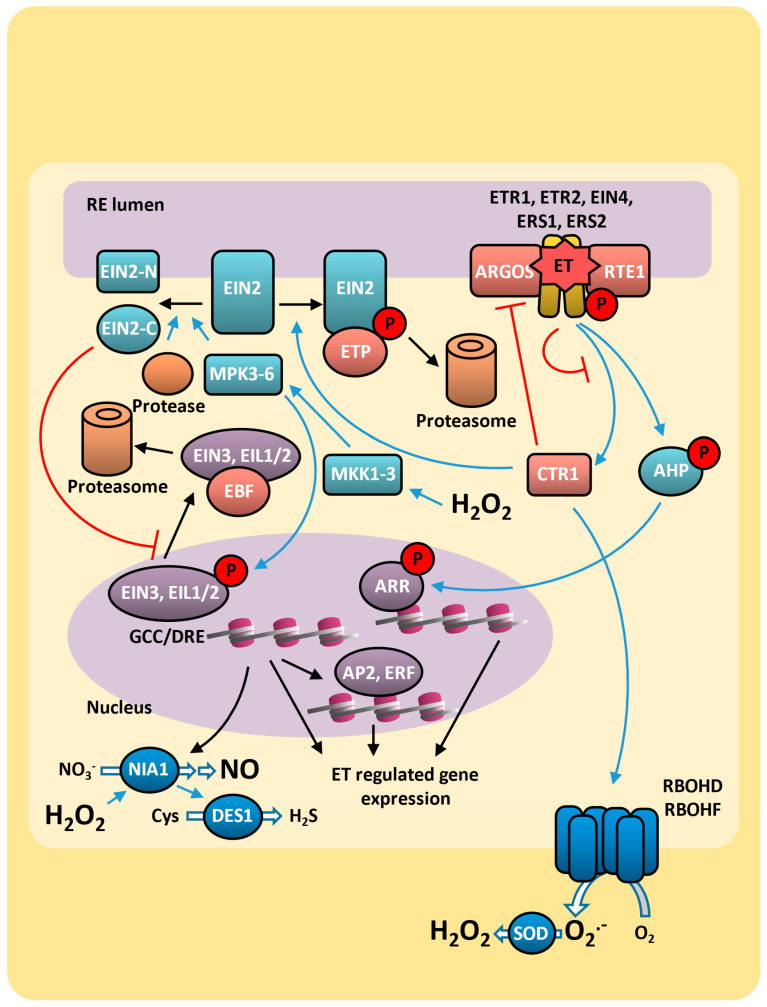
Ethylene signaling. Ethylene (ET) regulates key processes in plants, such as fruit ripening, leaf abscission, senescence, and stress responses. Ethylene signaling begins when ethylene binds to its receptors, including ETR1, ETR2, EIN4, ERS1, and ERS2, located on the endoplasmic reticulum (RE) membrane. The membrane protein RTE1 modulates receptor sensitivity, while ARGOS fine-tunes the strength of the signal. In the absence of ethylene, these receptors activate the negative regulator CTR1 that suppresses downstream signaling by inhibiting EIN2 by proteasome-mediated degradation. Upon ethylene perception, CTR1 is inactivated, allowing EIN2 to be cleaved. The C-terminal fragment of EIN2 translocate to the nucleus, where it stabilizes the transcription factors EIN3, EIL1, and EIL2 by preventing their degradation via the EBF proteins and proteasome. Stabilized EIN3 and EILs activate the transcription of ethylene-regulated genes, including those of the AP2/ERF family, which control several ethylene-mediated responses such as stress adaptation and developmental processes. NO produced by NIA1 and hydrogen sulfide synthesized by DES1 further modulate the ethylene signaling. The MAP kinases MPK3 and MPK6 also amplify ethylene responses. Additionally, AHP and ARR, components of the cytokinin signaling, interact with the ethylene signaling network. These proteins not only integrate ethylene signaling with other hormonal pathways but also regulate the expression of ethylene-responsive genes, influencing physiological responses such as growth regulation and stress adaptation. Ethylene signaling can also influence ROS production via the NADPH oxidases RBOHD and RBOHF, further modulating cellular responses. The hormonal receptor is indicated in yellow, while activators of the signaling are shown in blue and inhibitors in salmon. Transcription factors are in purple, and enzymes involved in ROS metabolism are in dark blue. Black lines with arrows indicate induction, blue lines with arrows indicate activation of phytohormone signaling, whereas red lines with bars represent inhibition.

The classic ethylene signal transduction is triggered by gas interaction with endoplasmic reticulum (ER) membrane receptors, including ETHYLENE RESPONSE 1 (ETR1), ETR2, ETHYLENE-INSENSITIVE 4 (EIN4), ETHYLENE RESPONSE SENSOR 1 (ERS1), and ERS2 [67], and the modulation of CTR1 (CONSTITUTIVE TRIPLE RESPONSE 1; EC 2.7.11.1) activity to regulate the expression of several genes (Figure 2).

The ethylene receptors are active in the absence of ethylene and constitutively activate the kinase activity of CTR1 [68], which is a Raf-like kinase able to phosphorylate the C-terminal domain of EIN2 (ETHYLENE-INSENSITIVE 2), which is also present in the ER membrane, leading its degradation by proteolysis. The EIN2 C-terminal domain previously phosphorylated by CTR1 interacts with ETP1 and ETP2 (EIN2 targeting proteins), which have F-box domains and are targeted for ubiquitination and degradation by the 26S proteasome [69]. The receptors activity can be positively regulated by complex formation with RTE1 (REVERSION TO ETHYLENE SENSITIVITY 1) and ARGOS (auxin-regulated gene involved in organ size) proteins, considered as negative regulators of ethylene sensitivity [70,71,72,73,74]. The active ethylene receptor biogenesis and the ethylene sensibility are also regulated by the availability of the copper cofactor, which is provided by the copper transporter RESPONSIVE-TO-ANTAGONIST 1 (RAN1), located in the ER membrane [75,76]. Upon binding with ethylene, the ethylene receptors undergo conformational changes, impairing the activation of CTR1. Consequently, instead of being phosphorylated, the EIN2 C-terminal domain is cleaved from the ER membrane by a protease enzyme and transported to the nucleus, thereby initiating ethylene signaling (Figure 2).

In the nucleus, EIN2 stimulates EIN3/EIL (ETHYLENE-INSENSITIVE 3; EIN3-like) transcription factors activity by repressing EBF (EIN3 binding F-box protein), which constitutively binds to EIN3/EIL, targeting it to ubiquitination and degradation [77]. Thus, under ethylene response, EIN3/EIL regulates the expression of ethylene-response genes by binding to the specific cis-acting AGCCGCC motifs (GCC box) and dehydration-responsive element (DRE) [78]. This pathway includes the ERF/AP2 transcription factor superfamily [79,80,81,82] that regulates the molecular response to different stresses, such as drought, salt, heat, and pathogen attack [78]. Interestingly, the EBF mRNA levels can be regulated by the exoribonuclease 5′-3′ EIN5 (ETHYLENE-INSENSITIVE 5, EC 3.1.1.3), another positive regulator of ethylene response, which promotes EBF mRNA decrease, thereby contributing to prevent EIN3/EIL proteolysis [83].

In addition to the canonical CTR1-dependent pathway, the ethylene signal also operates through noncanonical pathways. The EIN3/EIL transcription factors, for example, can be activated by alternative pathways, which involves a phosphorylation cascade of kinase proteins: MKK4-5-9 → MPK3-6 [83,84,85]. Once activated, MPK3-6 phosphorylates EIN3/EIL, preventing their interaction with the F-box protein EBF (EIN3 binding F-box protein). As a result, these factors accumulate in the nucleus and interact with target gene promoters, initiating various ethylene responses [85]. (Figure 2).

The noncanonical pathways also allow the ethylene receptors to interact with components of the cytokinin signaling [72,86,87,88,89]. The ETR1, a canonical histidine kinase, can directly interact with the histidine-containing phosphotransferase protein 1 (AHP1 in arabidopsis) through its C-terminus domain [87,90]. AHP1 is also phosphorylated by the cytokinin receptors by a phosphor-relay pathway and transfers the phosphate group to the response regulator proteins (ARR family in arabidopsis) that function as transcription factors during the cytokinin response [91]. Previous work demonstrated that the arabidopsis arr1 mutant showed reduced ethylene sensitivity in the regulation of root apical meristem size [92]. Indeed, ETR1 histidine kinase activity integrates ethylene and cytokinin signaling, inhibiting root growth [89]; nevertheless, this mechanism is not fully understood.

### 3.3. Ethylene and ROS on Drought Response

Under stress conditions, ethylene can elicit various responses, including the upregulation of antioxidative enzymes to scavenge ROS and alleviate oxidative stress as well as adjustments in plant physiology. During drought and salt stress, ethylene is known to decrease stomatal aperture, stimulate the growth of adventitious roots. and promote leaf senescence. These adjustments have a direct impact on plant stress response: (i) the stomatal closure stops water loss, (ii) the increased adventitious roots aid water absorption, and (iii) senescence may lead to the redistribution of nutrients in the sink organs [93]. Furthermore, under drought conditions, ethylene has been demonstrated to induce osmolyte biosynthesis [94] and leaf abscission, thereby reducing water loss [48,95]. The drought response mediated by ethylene can be highly regulated by hydrogen peroxide production via RBOHF (respiratory burst oxidase homolog protein F), which is inhibited by CTR1 [96]. In the presence of ethylene, CTR1 is deactivated, thus triggering the RBOHF-dependent hydrogen peroxide production in a mechanism mediated by Gα protein [97]. The increased hydrogen peroxide in guard cells also contributes to the cleavage and nuclear localization of EIN2 through the activation of MKK1/3–MPK3/6 signaling cascade [96]. This pathway induces the expression of nitrate reductase *NIA1*, which is also activated by hydrogen peroxide, contributing to the increase in NO synthesis. The NO, in turn, activates SLAC1 through both H_2_S-dependent and -independent pathways, leading to stomal closure [98]. Indeed, ethylene-induced NO synthesis activates L-cysteine desulfhydrase (DES1; EC 4.4.1.28), inducing H_2_S production and stomata closure [99]. Despite this evidence, ethylene is also able to inhibit the ABA-induced stomatal closure or to reduce stomatal sensitivity to stresses in different species [98,100]. These data suggest that while ABA is considered to act as the key regulator of stomatal closure under abiotic stress, ethylene can act as either a positive or negative regulator [101]. Thus, the mechanism by which ethylene regulates stomatal behavior during drought stress as well as the ethylene–ABA interaction pathway is complex and seem to be strongly dependent on their endogenous levels. This dual role during drought stress has led to ethylene and ABA being labeled “Foes or Friends” [101].

The ability of ethylene to either induce or inhibit stomatal closure during drought stress can be partially elucidated by the roles of its receptors. Among the five ethylene receptors described, only ETR1, EIN4, and ERS1 are involved in ethylene-induced stomatal closure, whereas ETR2 and ERS2 receptors may be involved in other ethylene-induced processes [97,102].

## 4. Salicylic Acid

Salicylic acid is a key signal molecule in regulating the activation of local and systemic defense responses against pathogens infections through the induction of defense genes [103,104,105]. Despite this, salicylic acid was first reported as a signaling molecule in plants, regulating the thermogenesis phenomenon in reproductive organs of gymnosperms and angiosperms [106,107]. Additionally, salicylic acid plays a regulatory role in abiotic stresses, like heat, cold, salinity, and drought [108,109,110], and influences plant growth and development by regulating various processes, including photosynthesis, respiration, vegetative growth, seed germination, flowering, and senescence [111,112,113].

### 4.1. Salicylic Acid Metabolism

Salicylic acid is a phenolic compound synthetized in plants via the isochorismate synthase (ICS, EC 5.4.4.2) or the phenylalanine ammonia-lyase (PAL, EC 4.3.1.5) pathways. Pathogen-induced salicylic acid is mainly synthesized via the ICS pathway in chloroplasts, which is mediated by three classes of enzymes: (i) ICS1, also known as SID2 (SALICYLIC ACID INDUCTION DEFICIENT 2) [114]; (ii) AtGH3.12 (also called AtPBS3 or avrPphB SUSCEPTIBLE 3), a family of cytosolic acyl adenylase proteins responsible for the conjugation of glutamate to isochorismate (IC) to form IC-glutamate; and (iii) EPS1 (ENHANCED PSEUDOMONAS SUSCEPTIBILITY 1), a BAHD acyltraferase-like protein responsible for the conversion of IC-glutamate to salicylic acid. This last step can also occur spontaneously [115,116,117]. Although the ICS pathway is fully elucidated, all three biosynthetic genes in this pathway, *ICS1*, *GH3.12*, and *EPS1*, are only found in the Brassicaceae family [116,118,119], while the PAL pathway is part of the phenylpropanoid metabolism and appears to be ubiquitous [117].

### 4.2. Salicylic Acid Signaling

**Figure 3:**
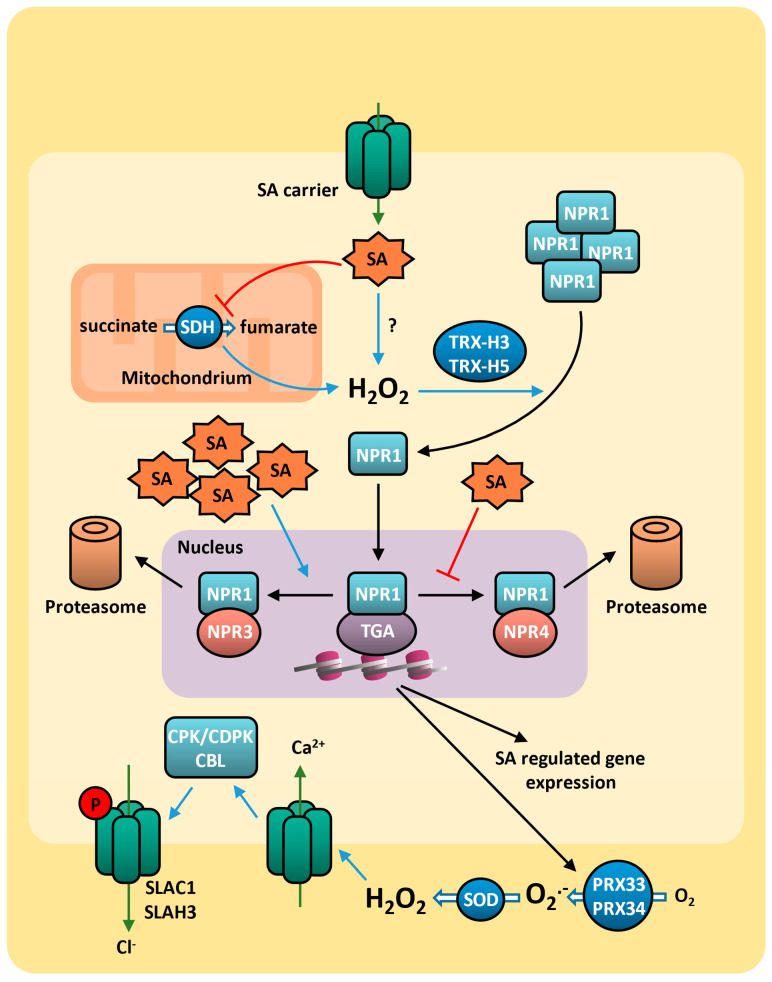
Salicylic acid signaling. Salicylic acid (SA)-mediated signaling is regulated by a complex mechanism involving the proteins NPR1, NPR3, and NPR4. In the absence of SA, NPR1 remains oligomerized in the cytosol. The monomerization of NPR1 is mediated by reactive oxygen species (ROS) produced by different mechanisms, which promote the reduction of disulfide bonds through the thioredoxins TRX-H3 and TRX-H5. Once monomerized, NPR1 translocate to the nucleus, where it regulates transcription factors involved in the salicylic acid response, such as TGA, activating the expression of defense genes like PR1. Additionally, PRX33 and PRX34 are induced by salicylic acid, increasing ROS production, which activates calcium channels and contributes to stomatal closure. It has been demonstrated that salicylic acid acts directly in the mitochondria, regulating ROS generation by the succinate dehydrogenase complex (SDH), contributing to the redox changes necessary for salicylic acid response. NPR3 and NPR4 act as SA receptors that regulate NPR1 activity; at high concentrations of salicylic acid, NPR3 and NPR4 direct NPR1 for degradation via the proteasome, thus controlling the plant’s defense response, particularly systemic acquired resistance, and modulates processes such as stress signaling, growth, and development. The activators of the signaling are shown in blue and inhibitors in salmon. Membrane channels/transporters are represented in green, transcription factors in purple, and enzymes involved in ROS metabolism in dark blue. Black lines with arrows indicate induction, blue lines with arrows indicate activation of phytohormone signaling, red lines with bars represent inhibition, and green lines with arrows indicate transmembrane transport. The step not yet confirmed is labeled with “?”.

Given the diverse roles of salicylic acid in environmental stress responses, it is likely that plants possess multiple salicylic acid receptors, and the protein NPR1 (NONEXPRESSOR OF PATHOGENESIS-RELATED GENES 1) is a redox-sensitive master regulator of salicylic acid signaling [120]. In the absence of salicylic acid, NPR1 is found oligomerized in cytosol. During stress response, when salicylic acid synthesis is induced, there is a change in the redox state at the cytoplasm, leading to the monomerization of NPR1 proteins. In its monomeric form, NPR1 is translocated to the nucleus, where it activates the expression of salicylic acid-responsive genes, including *PR* genes [121] and those involved in the induction of the hypersensitive response (HR) [103,122] (Figure 3).

The redox modification of cysteine residues in the protein–protein interaction and the cryptic trans-coactivation domains of NPR1 is essential for its monomerization [120,123,124]. Indeed, in arabidopsis and rice (*Oryza sativa*), mutations in conserved Cys82 and Cys216 of NPR1 resulted in this constitutive monomerization and nuclear localization of NPR1, resulting in the activation of defense genes expression [125]. Salicylic acid-induced AtNPR1 monomerization and nuclear import is catalyzed by thioredoxins AtTRXh3 and AtTRXh5, while in rice and tobacco, these processes are mediated by the orthologs OsTRXh2 and NtTRX1 [126,127]. In the absence of salicylic acid, NPR1 is also subject to degradation via the 26S proteasome [128], indicating that the regulation of NPR1 protein level is essential to salicylic acid response. NPR1 degradation is dependent on its interaction with NPR3 and NPR4 proteins, which have low and high affinity to salicylic acid, respectively [129]. While salicylic acid disrupts NPR1–NPR4 interaction, it facilitates NPR1–NPR3 interaction, creating an optimal NPR1 concentration gradient to regulate the salicylic acid response. In the absence or low concentration of salicylic acid, NPR4 mediates NPR1 degradation, whereas excessive salicylic acid levels prevent NPR1 accumulation by enhancing its interaction with NPR3 [130] (Figure 3). Thus, NPR1-mediated signaling is active only at intermediate salicylic acid levels. This model aligns with observations that NPR1 accumulates in regions adjacent to the pathogen infection sites, whereas at the infection site itself, the salicylic acid levels are even higher.

The salicylic acid response is mainly mediated by the upregulation of *pathogenesis-related* (PR) genes. The PR proteins have ~5–43 kDa and are thermostable, protease-resistant, and ubiquitously expressed, representing up to 10% of the total protein in leaves [131,132]. The PR proteins form at least 17 families, with distinct structure and function triggered by different pathogens [133,134]. The response to fungal attack, for example, involves PR1 (CAP-domain proteins), PR2 (β-1,3-glucanases), PR3 (chitinases), PR4 (Barwin-domain proteins), PR5 (thaumatin-like), PR12 (antimicrobial defensins), and PR17 (NtPRp27-like proteins) [132,134,135,136]. Additionally, salicylic acid positively regulates the biosynthesis of different defense molecules, such as flavonoid and phytoalexins [137]. This regulation is mainly mediated by NPR1 that regulates the transcription of salicylic acid-responsive genes mainly through its interactions with TGA (TGACG-binding) transcription factors, a subclass of the basic leucine zipper (bZIP) transcription factor family [138] (Figure 3). Besides the functional activation of transcription factors through complex formation, NPR1 also controls the expression of transcription factors genes, essential for salicylic acid-mediated transcriptional reprogramming, such as *WRKY* [139,140].

Although the salicylic acid signaling has not been fully characterized, it is known that salicylic acid regulates ROS production through mitochondrial respiration [141] and various enzymes related to ROS metabolism, such as CAT [142] and peroxidases (POD) [143]. At high concentrations, salicylic acid inhibits mitochondrial succinate dehydrogenase (SDH, EC: 1.3.5.1) and the respiration in tobacco (*Nicotiana tabacum*) cells [143] and in arabidopsis isolated mitochondria [144,145,146] (Figure 3). Conversely, at lower concentrations, salicylic acid induces SDH activity and mitochondrial oxygen consumption [141,145]. These findings suggest that salicylic acid signaling regulates mitochondrial respiration, leading to differential patterns of respiration and ROS production during the plant stress response. Indeed, a point mutation in SDH1-1 subunit (*disrupted stress response 1- dsr1*), which lowers SDH activity, results in decreased mitochondrial ROS production and impairs the stress response mediated by salicylic acid [147].

### 4.3. Salicylic Acid and ROS on Drought Response

Salicylic acid is also involved in abiotic stress tolerance [109,148], as it is increased under drought stress [149]. Salicylic acid induces stomatal closure [150,151] and decreases CO_2_ assimilation [152], inhibiting water loss and photosynthesis. Indeed, drought-induced ROS generation mediates salicylic acid-induced stomatal closure [130,153]. While plasma membrane NAD(P)H oxidases are the leading producers of ROS in guard cells induced by ABA signaling [30], salicyl-hydroxamic acid (SHAM)-sensitive peroxidases (PRX) are major ROS sources in guard cells induced by salicylic acid signaling [154]. The apoplastic PRX33 and PRX34, which are preferentially expressed in guard cells [155], are deeply involved in hydrogen peroxide production in response to various stimuli (including fungi, bacteria, and flg22), leading to stomatal closure [156,157,158,159,160]. Thus, the ROS generated by salicylic acid integrate into the ABA-induced stomatal closure pathway, activating calcium channels and inducing calcium influx [161] (Figure 3). Notably, salicylic acid-mediated ROS production and stomatal closure are not affected by NADPH oxidase inhibitor DPI (diphenyleneiodonium) or in the *rbohd*/*rbohf* double mutant [151].

Salicylic acid also regulates stomatal closure by increasing ethylene synthesis via induction of ACS2, ACS6, and ACS11 [162,163]. Indeed, ethylene biosynthetic inhibitors and mutations in ethylene-signaling genes (*ETR1*, *EIN2*, and *EIN3*) can inhibit stomatal closure induced by salicylic acid, which further suggests ethylene function downstream of salicylic acid to induce stomatal closure during drought [163]. Considering all these data together, ethylene and salicylic acid signaling may complement each other to induce stomatal closure during drought when ABA signaling is not sufficient [98].

## 5. Jasmonic Acid

Jasmonic acid acts both as a growth regulator and as a crucial signaling molecule in plant defense mechanisms [164]. Jasmonic acid orchestrates plant development and responds to a spectrum of biotic stresses, including physical injury, pathogen attacks, and insect predation, as well as numerous abiotic stresses, like low temperature, drought, excessive salinity, and UV radiation [165,166]. However, the intricate jasmonic acid-mediated signaling network, vital for safeguarding against external threats, can sometimes inhibit plant growth and development due to its over-activation by various regulatory factors [167].

### 5.1. Jasmonic Acid Metabolism

In response to developmental and environmental cues, plants rapidly produce a substantial increase in jasmonic acid, with is synthesized from α-linolenic acid through a series of reactions that occur sequentially in plastids and peroxisomes. Jasmonic acid–isoleucine conjugate (JA-Ile), the most biologically active component in jasmonate cascade, is synthesized by an ATP-dependent jasmonate–amino acid amidase encoded by *JAR1* (*JASMONATE RESISTANT 1*) [168,169,170,171]. On the other hand, the ω-oxidation pathway, involving members of CYP94B3, mediates the turnover and inactivation of JA-Ile, which is converted to 12-hydroxy-JA-Ile (12-OH-JA-Ile) [172].

### 5.2. Jasmonic Acid Signaling

**Figure 4:**
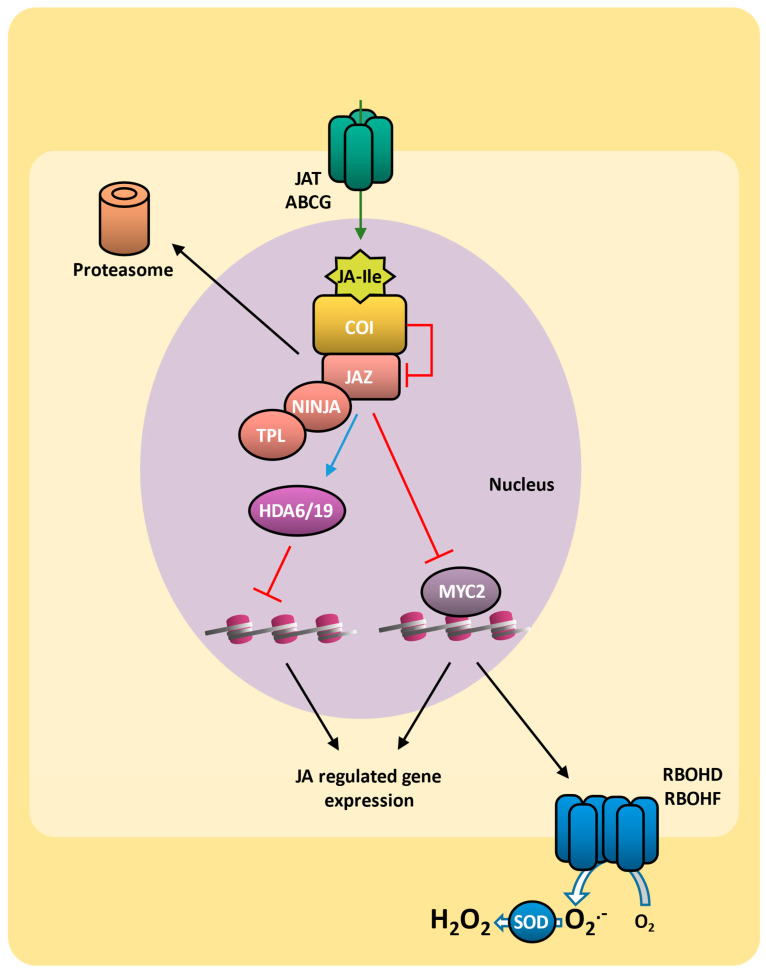
Jasmonate signaling. Jasmonates orchestrate plant defense against herbivores and pathogens while influencing processes such as reproductive development, secondary metabolite production, and adaptation to environmental challenges. Active jasmonate, in the form of jasmonoyl–isoleucine (JA-Ile), is transported to the cytoplasm by the ABCG and JAT transporters, where it binds to the COI1 receptor. This binding activates JA signaling by promoting the degradation of JAZ proteins via the proteasome. Under basal conditions, JAZ proteins suppress defense gene expression by interacting with the corepressor NINJA and TPL and recruiting deacetylases HDA6 and HDA19 to maintain chromatin in an inactive state. With the degradation of JAZ proteins, MYC transcription factors, particularly MYC2, are released, promoting the transcription of genes involved in stress response. This includes the activation of plasma membrane enzymes RBOHD and RBOHF, which contributes to ROS production. The hormonal receptor is indicated in yellow, while inhibitors in purple. Transporters are represented in green, transcription factors in purple, histone deacetylase in light purple, and enzymes involved in ROS metabolism in dark blue. Black lines with an arrow indicate induction, blue lines with arrow indicate activation of phytohormone signaling, red lines with bar represent inhibition, and green lines with arrow indicate transmembrane transport.

Jasmonic acid and JA-Ile subcellular distribution is controlled by JAT (JASMONATE TRANSPORTER), which is an ABC transporter (ABCG16 in arabidopsis) with an unexpected dual location at both the plasma membrane and nuclear envelope [173]. Jasmonic acid signaling is initiated by the hormone being perceived by the COI1 (CORONATINE-INSENSITIVE 1) nuclear receptor, which, upon jasmonate binding, interacts with the repressor JAZ (jasmonate-ZIM-domain) protein. In this context, COI1 binds to other proteins to form the Skp1/Cullin/F-box (SCF^COI1^) ubiquitin ligase complex, which ubiquitinates the JAZ protein, targeting it for degradation (Figure 4) [174,175]. In the absence of jasmonic acid, JAZ proteins recruit the protein TPL (TOPLESS) and the adaptor protein NINJA (Novel INteractor of JAZ) to form an effective transcriptional repressor complex [176] that inhibits various transcription factors, including MYC2 (MYELOCYTOMATOSIS 2). The MYC2 proteins are the main transcription factors in jasmonic acid signaling, exerting significant influence on the transcriptional reprogramming of several stress-responsive genes [167]. MED25 (Mediator 25), also known as PFT1 (PHYTOCHROME AND FLOWERING TIME 1), connects MYC2 with RNA polymerase II, thereby modulating gene transcription [177,178,179]. The JAZ–NINJA–TPL complex also recruits histone deacetylases HDA6 and HDA19, preventing the activation of jasmonate-responsive genes [177,180,181,182] (Figure 4).

Jasmonic acid plays an important role in plant biotic stress responses, triggered mainly by insects or pathogens. Jasmonic acid enhances the production of toxic proteins such as the defensive enzyme PPO (polyphenol oxidase), which affects the digestive physiology of herbivories, thereby protecting plants from herbivory [183,184]. Additionally, many secondary metabolites, such as tannins, phenols, flavonoids, and lignin, are induced by jasmonic acid and contribute to the resistance to biotic stress [185,186]. Through MYC2 activation, jasmonic acid regulates the accumulation of ROS through RBOHD and RBOHF [187], which act as a downstream signal following JAZ resulting in systemin-induced defense genes in response to wounding [188]. Like wounding, jasmonic acid also leads to a strong NO burst [189], which is involved in some jasmonic acid-mediated defense responses, inhibiting hydrogen peroxide accumulation [190,191].

Although salicylic acid regulates plant defense responses against some pathogens, salicylic acid and jasmonic acid signaling are antagonists [192]. While jasmonic acid-mediated defense is related to necrotrophy, the salicylic acid-mediated defense is related to hemibiotrophs [117]. The COI1 receptor regulates jasmonic acid-mediated inhibition of the salicylic acid pathway [193]. In a similar way, NPR1 not only controls salicylic acid signaling but antagonizes jasmonic acid signaling [194]. Jasmonic acid modulates the interaction between MYC2 and three NAC transcription factor genes (*ANAC019*, *ANAC055*, and *ANAC072*) to block the accumulation of salicylic acid by inhibiting the expression of genes involved in salicylic acid biosynthesis [195]. Additionally, MPK4 serves as a positive regulator of *GRX480* in the salicylic acid signaling but negatively regulates *MYC2* in the jasmonic acid signaling [196].

### 5.3. Jasmonic Acid and ROS on Drought Response

The activation of jasmonic acid-mediated signaling also contributes to plant tolerance to water stress [27]. Arabidopsis mutants of jasmonic acid signaling *coi1-2*, *jaz1*, and *myc2-2* exhibit drought sensitivity, indicating the importance of signal transduction mediated by jasmonic acid in drought response [197]. Additionally, during the response to drought in rice, OsJAZ proteins interact with the transcription factor OsbHLH148 and induce the expression of the DREB (DEHYDRATION-RESPONSIVE ELEMENT-BINDING) protein, contributing to drought tolerance [198]. During drought stress, jasmonic acid contributes to reducing stomata apertures [199]. Indeed, external application of jasmonic acid induces stomatal closure, triggering a robust response of plants to water stress [200]. This mechanism occurs with or without ABA contribution and is related to ROS production via RBOHD and RBOHF enzymes [199]. 12-OPDA (12-oxo-phytodienoic acid), one of the precursors of jasmonic acid, is also able to induce stomatal closure. Dry conditions prevent the conversion of 12-OPDAs into jasmonic acid, leading to 12-OPDA accumulation, which stimulates stomatal closure, either in conjunction with or independently of ABA, preventing water loss [15,201,202].

Jasmonic acid also boosts the activity of antioxidant enzymes such as superoxide dismutase (SOD), catalase (CAT), ascorbate peroxidase (APX), monodehydroascorbate reductase (MDHAR), dehydroascorbate reductase (DHAR), glutathione peroxidase (GPX), glutathione reductase (GR), glutathione S transferase (GST), and G-POD (guaiacol peroxidase) [203,204], providing protection against oxidative damage and increasing stress tolerance [205]. During drought stress, jasmonic acid-induced NO regulates the AsA-GSH cycle activity, contributing to the maintenance of AsA/DHA and GSH/GSSG ratios, protecting plant cells against oxidative damage [206].

## 6. Gibberellins

Gibberellins are tetracyclic diterpenoid carboxylic acids and play a crucial role in plant growth, regulating crucial aspects throughout both the vegetative and reproductive phases of a plant’s life cycle, such as seed germination, immature and mature stages of plant development, and flowering time. Additionally, gibberellins regulate the response to different abiotic stresses, including drought [15].

### 6.1. Gibberellins Metabolism

**Figure 5:**
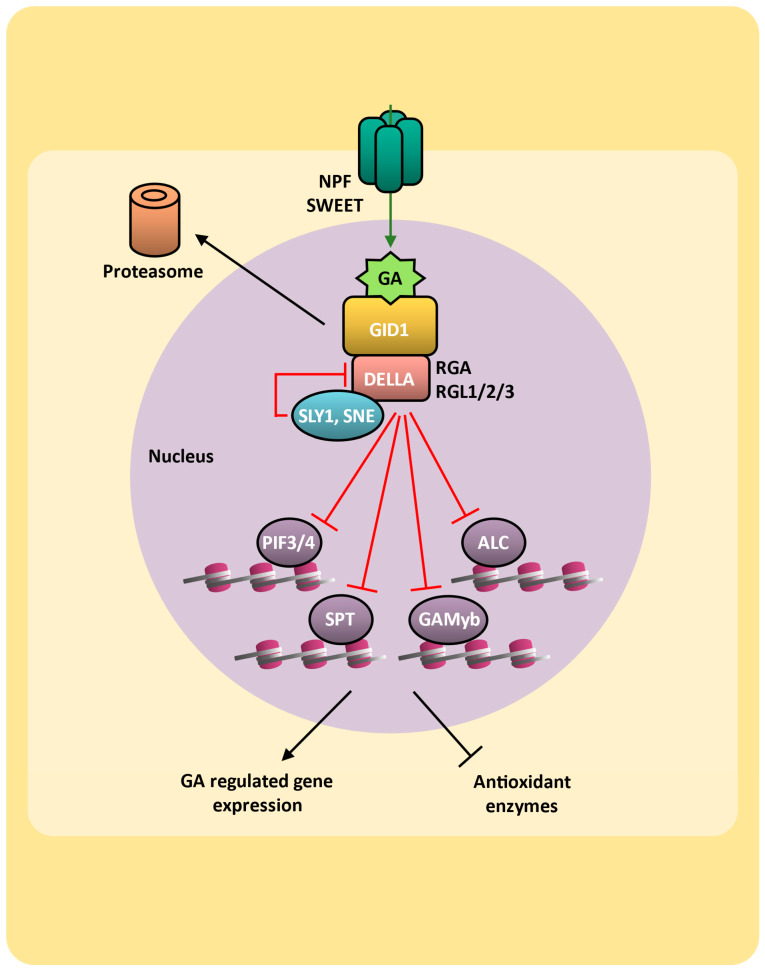
Gibberellin signaling. Gibberellin (GA) is transported across membranes by NPF and SWEET transporters. Once in the cytoplasm, GA binds to its receptor, GID1, forming a GA–GID1 complex. This complex interacts with DELLA proteins, key repressors of GA signaling. Upon binding, the GA–GID1 complex recruits the E3 ubiquitin ligase SLY1, which targets DELLA proteins for 26S proteasome degradation. DELLA degradation releases transcription factors such as PIF3, PIF4, SPT, ALC, and GAMyb, enabling them to activate genes involved in gibberellin response. Through this pathway, GA orchestrates key growth responses in plants, modulating developmental processes in response to internal and environmental signals. The hormonal receptor is indicated in yellow, while activators of the signaling are shown in blue and inhibitors in salmon. Transporters are represented in green, transcription factors in purple, and enzymes involved in ROS metabolism in dark blue. Black lines with arrows indicate induction, black line with bar indicates repression, red lines with bars represent inhibition, and green line with arrow indicates transmembrane transport.

Gibberellins are synthesized from geranylgeranyl diphosphate (GGDP) through the terpenes pathway. Among the 136 gibberellins structurally characterized, a few have been shown to be biologically active, such as GA_1_, GA_3_, GA_4_, and GA_7_ [207]. The non-biologically active GA_12_ is the first gibberellin in this biosynthesis route, and through the activities of different GA-oxidases, such as GA-20ox and GA-3ox (EC 1.14.11 and EC 1.14.11.15), it is converted into active gibberellins as well as other inactive intermediaries [208]. Gibberellin catabolism and deactivation occur mainly by oxidation reactions carried out by GA-13ox (EC 1.14.-.-) [209] and GA-2ox (EC 1.14.11.13) [210]. Alternatively, the formation of methyl esters by GAMT1 (gibberellin methyl transferase-1) is also able to inactivate gibberellins [211]. Gibberellins can be transported across the plasmatic membrane through transporters from SWEET (SUGAR WILL EVENTUALLY BE EXPORTED TRANSPORTER) [212] and NPF [213] (Figure 5).

### 6.2. Gibberellins Signaling

In the absence of gibberellin, the DELLA repressors proteins, such as SLR1 (SLENDER RICE 1) in rice and GAI (GIBBERELLIC ACID-INSENSITIVE), RGA (REPRESSOR-OF-ga1-3), RGL1 (RGA-LIKE 1), RGL2, and RGL3 in arabidopsis, act as primary suppressors of gibberellin hormonal responses based on the specific protein interaction partner [214]. DELLA proteins are recruited to target promoters by binding to transcription factors via the LHR1 subdomain [215]. Additionally, the DELLA–histone 2A interaction, via the PFYRE subdomain, is crucial for stabilizing the TF–DELLA–histone 2A complex at the target chromatin, mediating global transcriptional regulation [216].

Gibberellin signaling is initiated by its perception by the nuclear GID1b/c receptor (GIBBERELLIN-INSENSITIVE DWARF 1), leading to the ubiquitination of DELLA repressor protein through SCF (S-PHASE KINASE-ASSOCIATED PROTEIN 1/CULLIN 1/F-BOX)-type E3-ubiquitin ligase) complexes, resulting in its degradation via 26S proteasome and culminating in phytohormone response activation (Figure 5). In rice, DELLA ubiquitination is provided by SCFGID2 (SCF complex associated with the F-box protein subunit GID2) [217,218], while in arabidopsis, DELLA is ubiquitinated by SCFSLY1 or SCFSNE (SCF complexes associated with the F-box protein subunit SLEEPY1 or SNEEZY) [219,220,221,222,223]. Although DELLA proteins are typically inactivated by protein degradation, the gibberellin-induced interaction with the GID1 receptors is sufficient to inhibit DELLA activity when DELLA ubiquitylation and proteasomal degradation are blocked [214]. This mechanism is confirmed in rice *gid2* and arabidopsis *sly1* mutants, which show a comparatively mild phenotype and accumulate very high levels of DELLA proteins [224,225].

DELLAs interact and inhibit different transcription factors related to different aspects of plant development, such as the phytochrome-interacting factors PIF3 and PIF4, involved in photomorphogenesis [226,227]; ALC (ALCATRAZ) and SPT (SPATULA), repressors of cotyledon expansion [228,229]; and GAMyb (gibberellin-induced myb-like transcription factor) that regulates the expression of α-amylase, mobilizing the starch during seed germination [214,230] (Figure 5).

The role of ROS in gibberellin signaling appears to be more related to seed germination, as it was demonstrated that hydrogen peroxide accelerates the germination and stimulates the early growth of seedlings [231]. In aleurone cells of cereal seeds, gibberellin decreases the activities of antioxidant enzymes SOD, CAT, and APX, contributing to the increase in hydrogen peroxide levels [232], which are involved in programmed cell death (PCD) during seed germination [233]. The ROS accumulation and PCD induced by GA in aleurone cells are suppressed by ABA [234].

### 6.3. Gibberellins and ROS on Drought Response

In stress conditions, the bioactive form of gibberellin is inactivated due to the positive regulation of GA2ox6 and GA2ox7, resulting in 2β-hydroxylation, leading to the activation of the DELLA repressor [235]. The reduction in gibberellin levels and accumulation of DELLA has been associated with increased drought tolerance. Reduced gibberellin levels were shown to trigger the activation of various stress-related genes [236] as well as the accumulation of osmolytes [237] and enzymes that scavenge ROS [238], all of which are linked to drought tolerance. Drought tolerance in tomatoes (*Solanum lycopersicum*), for example, can be achieved by decreasing the expression of gibberellin biosynthesis genes through the action of DREB protein [239], by the loss of the *GID1* receptor gene [240], or by stable gain-of-function mutations in the *RGA* gene [241,242]. Similarly, transgenic tomatoes that overexpress arabidopsis *GAMT1*, responsible for methylating and inactivating gibberellin, exhibit reduced levels of gibberellin and increased drought tolerance [211]. Gibberellin signaling is an important regulator of cellular redox status, inhibiting the activity of various antioxidant enzymes [232]. Thus, the disruption of gibberellin during drought stress may play a key role in enhancing protection against oxidative stress, while ROS levels increase to fulfill their signaling functions.

## 7. Auxin

Auxin regulates cell division, elongation, tissue differentiation, embryogenesis, root formation, apical dominance, phyllotaxis, and tropic responses [243].

### 7.1. Auxin Metabolism

The indole-3-acetic acid (IAA) is the predominant naturally occurring auxin and is synthesized from the tryptophan precursor. Two steps compose the most important and well-characterized auxin biosynthesis pathway in plants, the TAA/YUC (tryptophan amino transferase/YUCCA) pathway. The amino acid tryptophan is first converted to indole-3-pyruvate (IPA) by the TAA (EC 2.6.1.99). Subsequently, the YUC-type flavin-containing monooxygenases (FMOs; EC 1.14.13.8) catalyze the production of IAA using IPA as a substrate [244], and this reaction seems to be the rate-limiting step in auxin biosynthesis [245]. IAA levels can be controlled mainly through IAA–amino acid conjugation by GH3 (Gretchen Hagen 3) or irreversible oxidation and degradation by DAO (dioxygenase for auxin oxidation) [246]. Auxin is synthesized at the shoot apex and transported by the mature phloem through a cell-to-cell transport mechanism named polar auxin transport [247]. Auxin flux in and out of the cell is mediated by AUX1/LAX (AUXIN RESISTANT1/LIKE AUX 1) permeases and PIN carrier proteins [248,249].

### 7.2. Auxin Signaling

**Figure 6:**
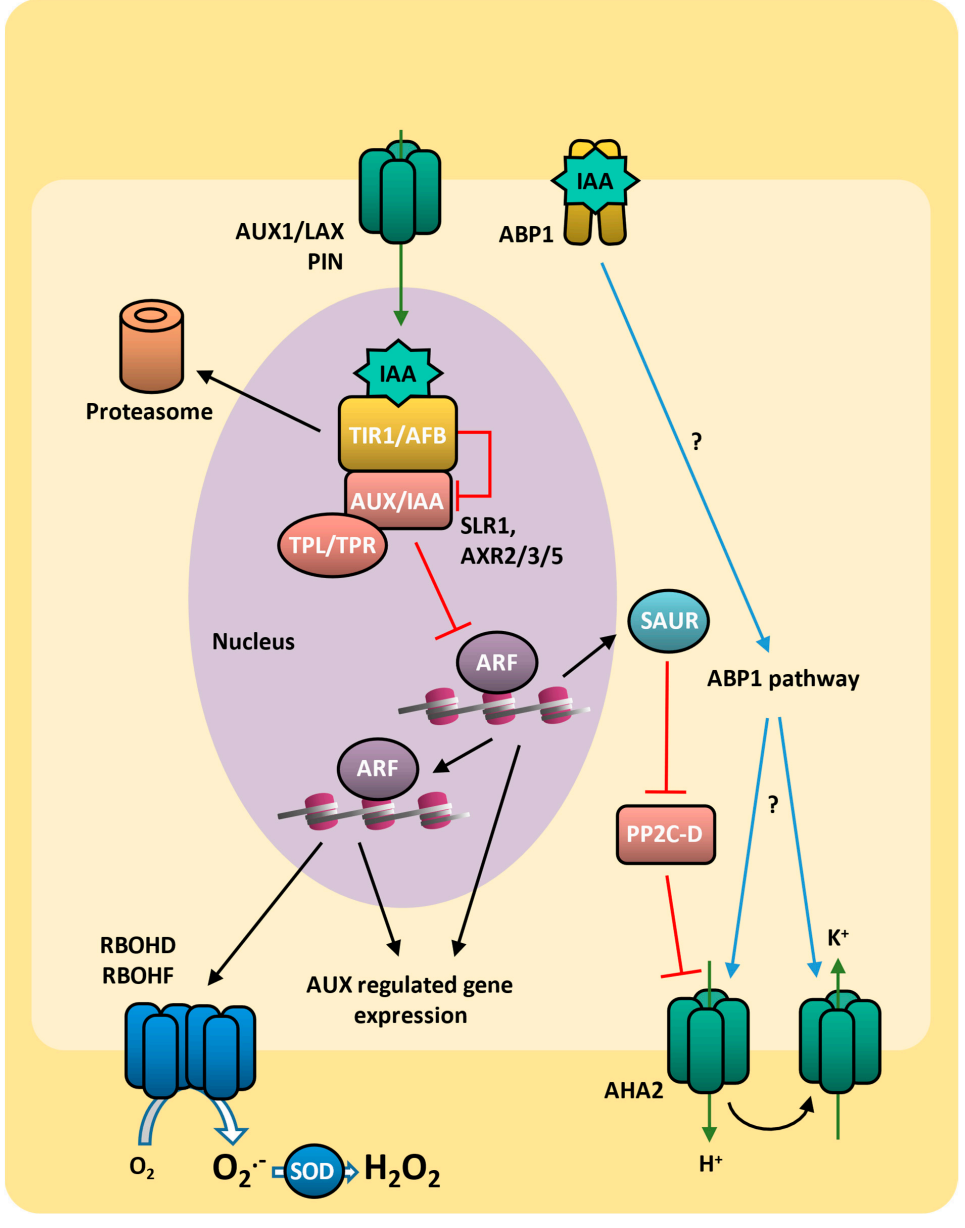
Auxin signaling. Auxins are central to plant development, controlling cell elongation, apical dominance, root initiation, and tropic responses to light and gravity. The indole-3-acetic acid (IAA) is the predominant naturally occurring auxin, and its transport is mediated by AUX1/LAX influx carriers and PIN efflux transporters. In the nucleus, the TIR1/AFB receptor complex, upon auxin binding, promotes the proteasome-mediated degradation of AUX/IAA repressor proteins. This releases ARF transcription factors from the AUX/IAA–TPL/TPR corepressor complex, allowing them to regulate auxin-responsive genes, including SAUR proteins and the NADH oxidases RBOHD and RBOHF. SAUR proteins inhibit PP2C-D phosphatases, leading to the activation of AHA2 proton pumps, which drive cell elongation. Reactive oxygen species (ROS), such as superoxide (O_2_^·−^), produced by RBOHD and RBOHF, are converted into hydrogen peroxide by superoxide dismutase (SOD), further influencing cellular processes. Although the pathway mediated by the ABP1 membrane receptor remains incompletely understood, it positively regulates AHA2 proton pumps and potassium channels, supporting auxin-driven responses. The hormonal receptor is indicated in yellow, while activators of the signaling are shown in blue and inhibitors in salmon. Membrane channels/transporters are represented in green, transcription factors in purple, and enzymes involved in ROS metabolism in dark blue. Black lines with arrow indicate induction, blue lines with arrows indicate activation of phytohormone signaling, red lines with bars represent inhibition, and green line with arrow indicates transmembrane transport. Steps not yet confirmed are labeled with “?”.

Auxin-mediated signaling is dependent on the AUX/IAA (AUXIN/IAA) inhibitory proteins such as SLR1 (SOLITARY-ROOT 1) [250], AXR2 (AUXIN-RESISTANT 2) [251], and AXR3 (AUXIN-RESISTANT 3) [252]. AUX/IAA proteins contain an N-terminal EAR (ETHYLENE-RESPONSIVE ELEMENT BINDING FACTOR-ASSOCIATED AMPHIPHILIC REPRESSION) motif and a C-terminal PB1 (PHOX AND BEM 1) domain. In the absence of auxin, AUX/IAA binds to ARF (AUXIN RESPONSE FACTOR) transcription factors through its PB1 domain and to TPL/TPR (TOPLESS/TOPLESS-RELATED) corepressor proteins by its EAR motif, resulting in the repression of ARF [253] (Figure 6).

The nuclear receptor TIR1/AFB (TRANSPORT INHIBITOR RESPONSE 1/AUXIN SIGNALING F-BOX), which is associated with SCF-type E3-ubiquitin ligases, recognizes auxin, so in the presence of the phytohormone TIR1/AFB, it binds to the AUX/IAA repressor protein, leading to its ubiquitination and proteolysis. This results in the release of the transcriptional activity of ARF transcription factors, leading to expression of downstream auxin-responsive genes [253,254]. ARFs bind directly to the auxin-responsive promoters by the AuxRE (auxin-responsive element), regulating their transcriptional activation or repression (Figure 6).

In addition to the Aux/IAA/SCF^TIR1/AFB^ pathway, AUX response is also mediated by ABP1 (auxin binding protein 1). The quest for auxin receptors has spanned decades, with the identification of ABP1 long before TIR1/AFB. Initial research identified an *abp1* mutant as the auxin receptor. Later works have led to uncertainty about ABP1’s role [255]. Recent re-analysis of *abp1* mutants has demonstrated a division between proponents of ABP1 and TIR1 as primary auxin receptors [256,257,258,259]. Recent studies have confirmed ABP1 as a crucial auxin receptor, particularly for vascular formation and regeneration, confirmed by the observation that *abp1* mutants show defects in auxin-induced vasculature [260]. ABP1 predominantly resides in the ER but is also secreted to the apoplast, where it binds auxin at the acidic pH. In the presence of auxin, the secreted ABP1 interacts with TMKs (TRANSMEMBRANE KINASES), forming the ABP1–TMK1 module that mediates rapid auxin-induced phosphorylation events [260,261], activating ion fluxes at the plasma membrane in response to auxin stimuli [262,263,264] and the MAPK cascade [265,266] (Figure 6). ABP1 also modulates the expression of AUX/IAA genes, indicating its broader role in influencing the Aux/IAA/SCF^TIR1/AFB^ pathway [267].

The two auxin receptors, ABP1 and TIR1, regulate multiple targets, with their actions tailored to the specific developmental context. ABP1 is critical for auxin regulation of the cell cycle, regulating the G1/S transition [268]. In fact, abnormal cell divisions occur within the suspensor and during the initial phases of embryo development in the arabidopsis mutant lacking *ABP1* [269]. Auxin also stimulates cell elongation by initiating an acidification process within the cell wall, mediated by the activation of plasma membrane AHA2 (autoinhibited H^+^-ATPase 2), which exports protons to the apoplast (Figure 6). The low pH in the apoplast triggers a cascade of reactions that facilitate the “loosening” of the cell wall structure. This loosening will allow the irreversible expansion of the cell wall, driven by turgor pressure, ultimately leading to cell elongation [270]. The activation of AHA2 also induces hyperpolarization of the membrane potential, resulting in the opening of voltage-dependent inward K^+^ channels [271,272]. The influx of K^+^ contributes to water uptake and turgor, allowing cell expansion. Moreover, besides directly stimulating their activity, auxin also prompts the expression of both AHA2 channel and K^+^ channel genes [272,273].

AHA2 activity is inhibited by its dephosphorylation via PP2C-D family of protein phosphatases, which are inhibited by SAUR (small auxin upregulated RNA) [274]. SAUR19 is induced by auxin and promotes cell expansion through binding to PP2C-D and releasing AHA2 activity [275]. AHA2, as well as K^+^ inward channels, are also triggered by ABP1 during the initial perception of auxin, but the mechanism remains elusive [270] (Figure 6).

Because auxin regulates growth by cell elongation or division, auxin tends to act downstream in the regulation of many growth processes, such as cotyledon growth, hypocotyl elongation, root elongation, and lateral root formation [270,276]. Additionally, auxin biosynthesis, transport, and signaling are required by the ABA inhibitory effect in seed germination. This was demonstrated in the mutants *yuc1 yuc6* [277], *pin2* [278], and *tir1* [279], which display resistance to the inhibitory effects of ABA on seed germination.

### 7.3. Auxin and ROS on Drought Response

Many works have highlighted the versatile involvement of auxins in enhancing plant tolerance to abiotic stresses [280]. Indeed, the overexpression of the auxin biosynthesis gene *YUC6* is associated with enhanced drought tolerance and distinct phenotypic changes in arabidopsis [281], while the overexpression of *OsGH3.2*, involved in salicylic acid biosynthesis in rice, results in reduced levels of auxin and more sensitivity to drought [282,283]. In arabidopsis submitted to drought stress, auxin accumulation can also be triggered by downregulation of miRNA167. This miRNA targets *IAR3* (IAA–ALANINE-RESISTANT 3) mRNA, which encodes an IAA–alanine hydrolase responsible for releasing active IAA from the IAA–alanine conjugate [284].

Auxin displays a crucial role in regulating genes involved in the response to water stress via *ARF* induction [285]. In arabidopsis, auxin positively regulates the expression of various stress-related genes, such as *RAB18* (RESPONSIVE TO ABA 18), RD22 (RESPONSIVE TO DEHYDRATION 22), *RD29A* (RESPONSIVE TO DESICCATION 29A), RD29B (RESPONSIVE TO DESICCATION 29B), *DREB2A*, and *DREB2B* [286]. While ABA signaling induces stomatal closure, auxin regulates both stomatal movement and development, reducing stomatal density [287]. Arabidopsis mutants defective in auxin biosynthesis, transport, or signaling exhibit increased stomatal index, thereby establishing a negative role of auxin in stomatal development [288,289,290]. In mesophyll, auxin-induced ARF inhibits the expression of STOMAGEN peptide, which promotes stomata development [290,291]. During drought, plants utilize hydrotropism, an adaptive mechanism involving directed root growth towards water sources, to cope with this challenge. In this condition, the drought-induced auxin accumulation regulates the root system architecture, optimizing water uptake efficiency [292].

Auxin also contributes to the regulation of ROS levels, which are notably increased during drought. The *RBOHs* NADH oxidases’ expression and ROS levels are induced by RSL4 (ROOT HAIR DEFECTIVE SIX-LIKE 4), which is activated by ARF transcription factor and binds to at least one root hair cis-acting element (RHE) region in the *RBOHC* and *RBOHJ* promoters [293]. Indeed, ROS triggered by auxin are directly implicated in the loosening of the cell wall and contribute to the process of cell elongation [294]. The drought-induced ROS production significantly disrupts auxin homeostasis in plants [6], influencing polar auxin transport by modulating the expression PIN proteins and affecting auxin conjugation [295]. Similarly, ROS contributes to the oxidative degradation of auxin through peroxidase activity, resulting in changes in auxin gradients and disruption in hormone-mediated signaling [296]. Additionally, under water stress conditions, auxin has a positive regulatory effect on antioxidant enzymes SOD CAT, POD, and GR. This regulatory action intensifies ROS detoxification, thereby contributing to an effective defense mechanism against oxidative stress [286].

## 8. Cytokinin

Cytokinins are a group of phytohormones that regulate shoot and root growth, cell division and proliferation, chloroplast and vascular development, bud differentiation, anti-aging, biomass distribution [297,298,299], photosynthesis and nutrient metabolism [300], leaf senescence [301], and maintenance of meristem function [302,303]. In plants, the most abundant cytokinins are adenine-type species, including common derivatives such as isopentenyl adenine (iP), trans-zeatin (tZ), and cis-zeatin (cZ) [304]. In higher plants, zeatin is the most abundant cytokinin existing in both tZ and cZ forms. The tZ form is active in all plant species, whereas cZ is active only in specific plants, such as rice [305].

### 8.1. Cytokinin Metabolism

Cytokinins are synthesized via two primary biosynthetic pathways. The first one is de novo synthesis, where 5-phosphate adenosine (AMP, ATP, and ADP) is converted into iP/tZ-type cytokinins by adenosine phosphate isopentenyltransferase (IPT, EC 2.5.1.27) and CYP735A1/CYP735A2. The second biosynthetic pathway involves tRNA-specific adenylate isopentenyltransferase (tRNA-IPT), which indirectly leads to the formation of cZ-type cytokinins [304,306]. Cytokinins are synthesized in various cell types in both roots and shoots, and they undergo both local and long-distance transport [307,308,309,310]. Although the mechanisms of cytokinins transport are not fully understood, potential efflux and influx transporters have been identified, such as ABCG14, involved in cytokinin efflux across the plasma membrane [91,311].

Cytokinins degradation is primarily catalyzed by cytokinin oxidase/dehydrogenase (CKX, EC 1.5.99.12), resulting in the release of free adenine or adenine nucleosides, leading to the complete inactivation of the cytokinin [307]. Additionally, cytokinin inactivation can occur through conjugation to sugars via glucosyltransferases [304]. CKX and glucosyltransferases are both induced by cytokinins to deactivate the cytokinin signal itself. These negative-feedback pathways illustrate the tight regulation of cytokinin pathway output, reflecting its profound effects on plant development and the frequent targeting of cytokinin signaling by pathogens [91].

### 8.2. Cytokinin Signaling

**Figure 7:**
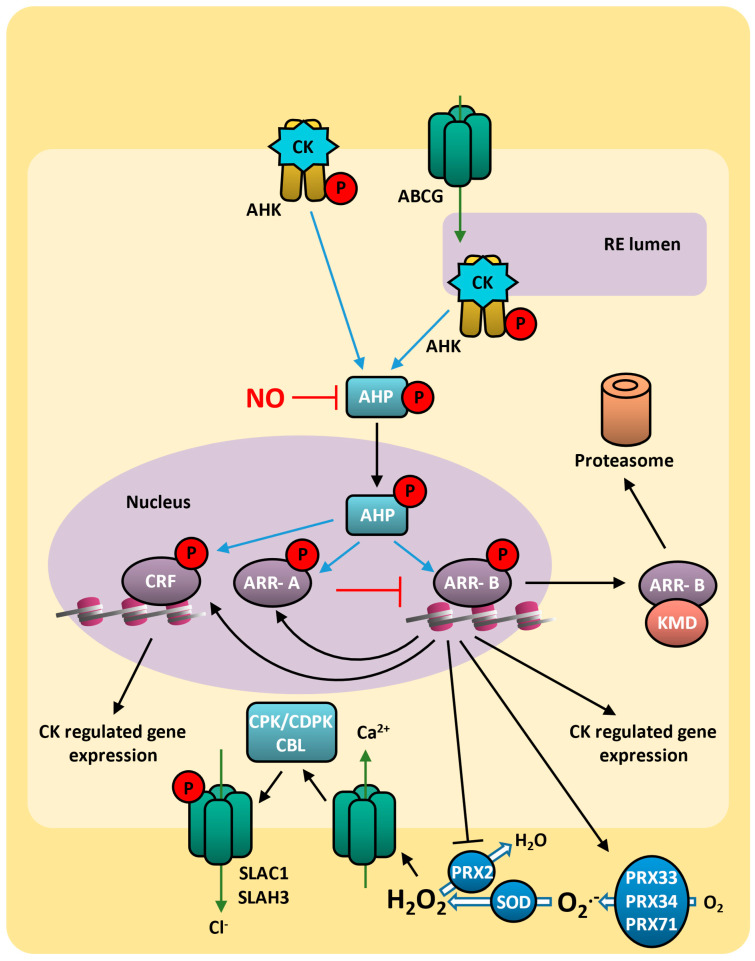
Cytokinin signaling. Cytokinin signaling is initiated when cytokinins (CK) bind to the AHK receptors, located in the plasma membrane or, alternatively, in the membrane of the endoplasmic reticulum (RE). When AHK is in the endoplasmic reticulum, cytokinins enter the cell via ABCG channels. Upon binding to AHK, the receptor activates a two-component signaling system, where AHK phosphorylates the response regulator AHP. The phosphorylated AHP then translocated to the nucleus, where it activates the transcription factors ARR-B. ARR-B induces the expression of several cytokinin-regulated genes, among them peroxiredoxins *PRX33*, *PRX54*, and *PRX71*, which produce superoxide. Superoxide is then converted into peroxide by superoxide dismutase (SOD). Additionally, ARR-B represses the expression of PRX2, a protein responsible for peroxide elimination, contributing to peroxide accumulation in the apoplast. In the apoplast, peroxide regulates and activates calcium channels, allowing calcium influx into the cytosol. This calcium influx activates calcium-dependent kinases (CPK/CDPK/CBL), which phosphorylate and activate chloride channels such as SLAC1 and SLAH3. ARR-B also induces the expression of *ARR-A*, which, through a negative-feedback mechanism, downregulates the activity of ARR-B. Furthermore, ARR-B is regulated by the protein KMD, which induces its degradation via the proteasome. Through these complex interactions, cytokinins regulate key developmental processes such as shoot and root development, lateral bud outgrowth, and leaf senescence. The hormonal receptor is indicated in yellow, while activators of the signaling are shown in blue and inhibitors in salmon. Membrane channels/transporters are represented in green, transcription factors in purple, and enzymes involved in ROS metabolism in dark blue. Black lines with arrow indicate induction, black lines with bar indicates repression, blue lines with arrow indicate activation of phytohormone signaling, red lines with bars represent inhibition, and green lines with arrow indicate transmembrane transport.

Cytokinins signal transduction is analogous to the bacterial two-component regulatory system, which consists of histidine kinases (HKs; sensing proteins) and reactive regulatory proteins (receiving proteins) [312]. Cytokinins bind to the histidine kinases receptors AHK2 (ARABIDOPSIS HISTIDINE KINASE 2), AHK3, AHK4/CRE1 (CYTOKININ RESPONSE 1), CKI1 (CYTOKININ-INDEPENDENT 1), and AHK5/CKI2 to form a dimer [313]. The conserved His residues in the N-terminal sensor-kinase domain are autophosphorylated, and the phosphate group is then transferred to a conserved Asp in the C-terminal receiver domain [314,315,316,317,318]. In general loss-of-function mutations of single receptors displayed only subtle effects on most of the phenotypes studied. However, in some cases, individual receptors were shown to mediate specific cytokinin activities [319]. The histidine kinase receptors are primarily localized at the ER membrane, suggesting that the site of cytokinin binding is within the ER lumen [320,321,322]. A small portion of HK receptors are also found at the plasma membrane [323] although there is ongoing debate regarding the extent to which these receptors are involved in mediating the cytokinin response [311] (Figure 7).

During cytokinins signaling, the phosphate group on the conserved Asp residue of the receptor is subsequently transferred to HPt (histidine phosphate transfer) proteins in the cytoplasm. Cytokinin receptors interact in vivo with all studied HPt proteins and vice versa [324] (Figure 7). The arabidopsis genome contains five AUTHENTIC HISTIDINE-CONTAINING PHOSPHOTRANSMITTER genes: *AHP1*, *AHP2*, *AHP3*, *AHP4*, and *AHP5*. The AHP proteins feature a conserved cysteine residue susceptible to S-nitrosylation by nitric oxide, both in vitro and in vivo, that inhibits their ability to receive phosphate from histidine kinases and transfer phosphate to response regulators [325]. Additionally, plants possess pseudo-histidine phosphotransferase proteins (PHPs), which are lacking in the histidine phosphorylation site [326,327,328,329]. PHPs act as negative regulators of cytokinin signaling in arabidopsis, influencing protoxylem differentiation [330,331], leaf phyllotaxy [332], and cell patterning during lateral root initiation [333].

In the nucleus, phosphorylated HPT transfers phosphate groups to ARR (Arabidopsis response regulator), thereby regulating the expression of cytokinin-responsive genes [334]. In cytokinins signaling, two types of ARR play distinct roles. Type-B ARRs are phosphorylated on an Asp residue in their receiver domain by AHPs, crucial for initiating the transcriptional response to cytokinin [335,336]. Turnover rates within the type-B RR family vary and are regulated in part by the KISS ME DEADLY (KMD) E3–ubiquitin ligase complex [337,338]. Conversely, type-A ARRs function as negative-feedback regulators of cytokinin signaling. They are transcriptionally induced by cytokinin through direct activation by type-B ARRs [339,340,341,342]. Among the targets of the transcriptional control mediated by type-B RRs is the transcription factor WUSCHEL, which regulates shoot meristem activity, thereby establishing a direct link between cytokinin signaling and the control of shoot growth and development [343,344,345,346].

The CRF (CYTOKININ RESPONSE FACTOR) group also acts as a side-branch of the two-component signaling system, being activated by HPt proteins [347]. The CRF is a non-monolithic group of AP2/ERF transcription factors found in most angiosperms and appears to be a target of ARR-Bs [348]. CRFs are recognized as key transcription factors in responding to abiotic stresses across various plant species, contributing to the trade-off between growth and stress response [349,350]. Moreover, several members of the CRF family from different plant species are induced by oxidative stress, which is considered one of the most critical consequences of abiotic stress [351,352].

Cytokinins frequently synergize with other hormones, particularly auxin, to coordinate cell division and differentiation. CRFs are capable of binding to elements found in the promoters of some *PIN* genes, controlling the expression of auxin transporters [353] and IAA distribution during root development [354,355,356,357]. Additionally, CRF2 was identified as being a downstream target of ARF5/MONOPTEROS, a key auxin-regulated transcriptional factor [358], suggesting that CRF acts as an important bridge between cytokinin and auxin signaling. In the shoot, cytokinins promote cell proliferation in apical and axillary meristems. Increased cytokinins levels, resulting from reduced expression of *CKX* genes, have been linked to enhanced grain yield in indica rice varieties [359], and disruption of *CKX* genes in arabidopsis leads to increased seed yield [360]. Additionally, cytokinins regulate leaf phyllotaxy [332], gynoecium development [361,362,363,364], female gametophyte development [365,366], and vascular cambial development [367,368]. In contrast, cytokinins inhibit root growth by promoting cell differentiation in the root apical meristem and by suppressing both lateral root initiation and primary root elongation [369,370,371,372,373,374]. Indeed, cytokinins determine the size of arabidopsis root meristems by regulating the rate of cell differentiation in meristems [375]. Overexpression of type-B *ARRs* can promote arabidopsis root growth, indicating that cytokinins participate in root development [376]. In rice, the overexpression of *OsIPT* disrupts root development [377]. Similarly, overexpression of *OsRR3* or *OsRR5* improves root growth and induces lateral root initiation [378]. Conversely, overexpressing the *Zea mays CKX* gene in *Nicotiana tabacum* reduces cytokinin levels, inhibits stem development, and enhances root growth [371].

Cytokinins also collaborate with salicylic acid-regulated plant immunity by the interaction between ARR2 and the SA response factor TGA3, which binds to the *PR1* promoter in arabidopsis during pathogen response [379]. Indeed, elevated cytokinin levels boost SA-mediated defense responses, providing greater resistance to infection by virulent oomycete pathogens [380] and arabidopsis transgenic plants overexpressing *AtCRF5* enhance resistance to *Pseudomonas syringae* pv. tomato DC3000 (Pst DC3000) [381]. Additionally, DELLA proteins, acting as central nodes in transmitting environmental cues, directly engage with type-B ARRs to co-activate gene targets crucial for root growth and photomorphogenesis [382].

### 8.3. Cytokinin and ROS on Drought Response

Cytokinins can also enhance resistance to adverse environmental factors [300]. Different studies suggest that cytokinins signaling is directly involved in drought stress responses. Exogenous cytokinin application was shown to improve the drought resistance of *Cucumis sativus* [383]. Similarly, the expression of the cytokinin biosynthetic enzyme IPT in tobacco enhances drought tolerance by delaying leaf senescence [384]. The balance between triggering leaf senescence and sustaining photosynthesis is crucial for drought tolerance and for maintaining crop yields under stress [385,386,387].

In arabidopsis, the CRF family may affect cytokinins signaling in the equilibrium between active photosynthesis and senescence. AtCRF6 acts as a negative regulator of leaf senescence. Its overexpression preserves chlorophyll (Chl) and delays leaf yellowing [388]. Similarly, AtCRF9 promotes chlorophyll retention in dark-induced senescence assays, maintaining photosynthesis during senescence [389]. In the opposite direction, the overexpression of *AtCRF1*, *AtCRF3*, and *AtCRF5* genes leads to early leaf senescence, and *crf1,3,5,6* multiple knockout delays this process [390], indicating that these genes act as positive regulators of leaf senescence. The overexpression of *AtCRF2* also accelerates senescence in rosette leaves [391], but it has also been shown to accelerate chloroplast division, a feature linked to enhanced photosynthetic activity [392,393].

Cytokinin is also demonstrated to play negative regulatory roles in drought stress response. In arabidopsis, cytokinin-deficient CKX-overexpressing plants show improved survival under drought stress [394]. Additionally, the receptors AHK2 and AHK3 [395]; the HPt proteins AHP2, AHP3, and AHP5 [396]; and the transcription factors ARR1, ARR10, and ARR12 [397] act as negative regulators of plant responses to drought. In tomato, cytokinins affect transpiration by regulating stomatal density and leaf size. Under drought stress, reduced levels of CKs lead to a decrease in the cell division rate, suppressing growth and reducing stomatal density. This, in turn, leads to lower transpiration rates, which enhances plant survival in drought conditions [398]. The cytokinin-mediated stomatal responses involve the apoplastic peroxidases PRX4, PRX33, PRX34, and PRX71 but not the NADPH oxidases RBOHD and RBOHF. While PRX2 acts as an ROS scavenger, PRX33, PRX34, and PRX71 contribute to ROS production and stomata closure. In guard cells, *PRX2* expression is repressed by ARR2, while *PRX33* is induced [160].

Stomata closure and ROS production induced by tZ are compromised in the histidine kinase *ahk3* and response regulators arr2 mutants, which are also defective in FLG22 (flagellin 22)-mediated ROS production. On the other hand, *IPT3* and *ARR2* overexpression lines show higher levels of ROS in guard cells and constitutive closed stomata phenotypes [160]. Importantly, *ost1-3* and *aba1-3* mutants, which are defective in FLG22-triggered stomatal closure [399,400], show wild-type stomatal closure in response to tZ treatment.

## 9. Brassinosteroids

Steroid hormones have been implicated in several aspects of animal growth and development. In plants, the steroid-like hormone brassinosteroid (BR) plays important roles in plant growth and development, being involved in the control of several developmental and physiological processes, such as cell elongation, cell division, vascular differentiation, senescence, flowering time control, male fertility, pollen development, seed size, photomorphogenesis, and resistance to biotic and abiotic stresses [401]. Despite having been named due to its original identification in *Brassica napus* pollen [402], the most active brassinosteroid, brassinolide (BL), has been found widely distributed in virtually all plant species [403].

### 9.1. Brassinosteroids Metabolism

Many of the important advances in the study of brassinosteroids come from a series of different genetic screenings. An important step was the identification of brassinosteroids biosynthesis mutants such as *de-etiolated 2* (*det2*) and *constitutive photomorphogenesis* (*cpd*). They were the first mutants identified in this biosynthesis pathway, and the genes encode, respectively, a 5α-reductase (EC 1.3.1.22) and a C23-steroid hydroxylase (EC 1.14.13.112) [404,405]. Both mutants have been identified in screening for mutants defective in hypocotyl elongation, and their phenotype can be reduced upon BL application. Brassinosteroids are produced in the cell, but several lines of evidence have shown that their perception occurs at the plasma membrane by a receptor complex that initiates the signaling [406]. Due to their polar nature, the bioactive brassinosteroids might be exported out the cells, but the mechanism by which this transportation occurs was previously unknown. Very recently, an arabidopsis ABC transporter protein (ABCB19) was identified as a functional brassinosteroid exporter [407].

### 9.2. Brassinosteroids Signaling

A deeper comprehension of the hormone’s perception and function comes from the identification of arabidopsis brassinosteroid signaling mutants. These mutants usually display dwarf phenotypes unable to respond to exogenous BL treatment. Brassinosteroids signaling is mediated by BR1 (BRASSINOSTEROID-INSENSITIVE 1) receptor, a membrane-bound receptor serine/threonine kinase protein with extracellular leucine-rich repeats [408,409,410]. Later, similar receptors were isolated and characterized in several other species, such as rice [411], tomato [412], pea (*Pisum sativum*) [413], barley (*Hordeum vulgare*) [414], currant tomato (*Solanum pimpinellifolium*) [415], petunia (*Petunia hybrida*) [416], wild tobacco (*Nicotiana attenuate*) [417], *Brachypodium distachyon* [418], maize (*Zea mays*) [419], and alfalfa (*Medicago truncatula*) [420]. The arabidopsis genome encodes three other BRI1-related genes. *BRL1* and *BRL3* but not *BRL2* encode functional brassinosteroid receptors that bind BL with high affinity. These genes display different tissue-specific expressions. *BRI1* is expressed ubiquitously in growing cells, while *BRL1* and *BRL3* expression are restricted to non-overlapping regions of the vascular tissue [421]. Similar to arabidopsis, *BRI1*-related genes have been also described in other plant species [411,414,419].

**Figure 8:**
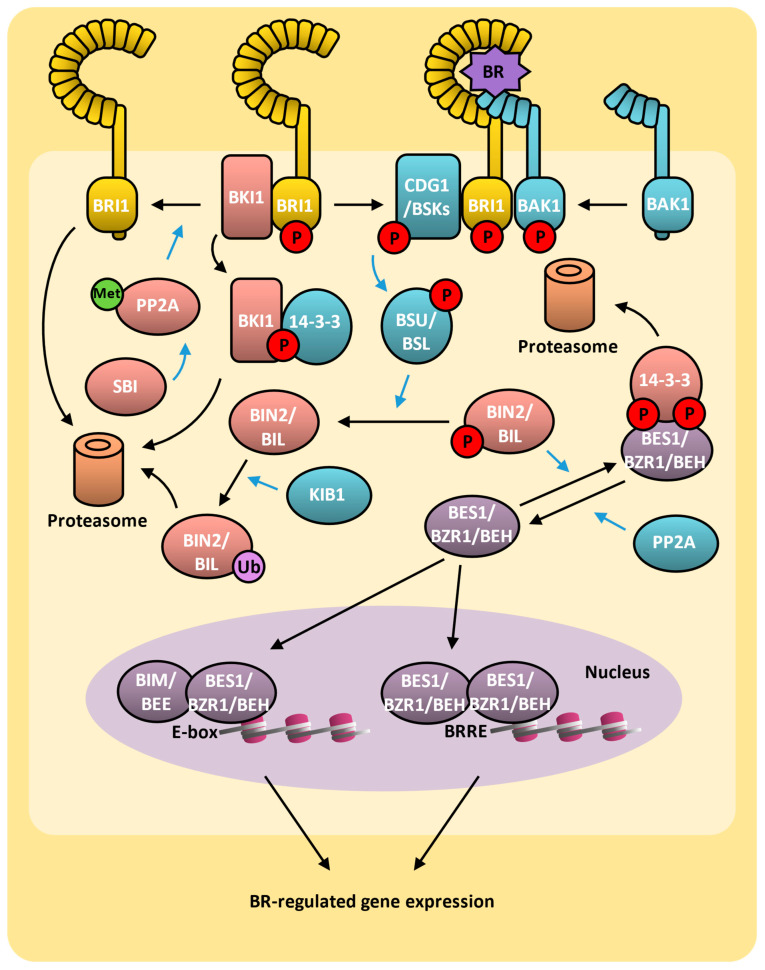
Brassinosteroids signaling. In the absence of brassinosteroids (BRs), the BRI1 receptor in the plasma membrane associates with BKI1, keeping the receptor inactive. This association is disrupted when BKI1 is phosphorylated, causing it to bind to 14-3-3 proteins, leading to its degradation via the proteasome. Additionally, BRI1 can be dephosphorylated by a type of PP2A phosphatase, which induces receptor proteasome degradation. PP2A are activated by the SBI1 methylase. Upon BR binding, BRI1 forms a complex with the co-receptor BAK1, triggering a kinase cascade that starts with the phosphorylation of CDG1/BSKs. These kinases phosphorylate the phosphatase BSU/BSL, which dephosphorylates the BIN2/BIL kinases. Once BIN2/BIL is dephosphorylated, it is ubiquitinated by KIB1 and degraded. BIN2/BIL is responsible for phosphorylating key transcription factors BES1/BZR1/BEH, which regulate the BR response. In their phosphorylated form, these transcription factors bind to 14-3-3 proteins, leading to their degradation and inhibiting BR signaling. When BIN2/BIL are inhibited or degraded, BES1/BZR1/BEH are no longer phosphorylated or can be dephosphorylated by PP2A. Unphosphorylated forms of BES1/BZR1/BEH are active and translocated to the nucleus to regulate gene expression. As homodimers, these transcription factors bind to BRRE elements, and as heterodimers with bHLH transcription factors like BIM and BEE, they bind to E-box elements, further controlling BR-responsive gene regulation. This pathway orchestrates key developmental processes, including cell elongation, growth, stress responses, and gene expression modulation in plants. The hormonal receptor is indicated in yellow, while activators of the signaling are shown in blue, inhibitors in salmon, and transcription factors in purple. Black lines with arrows indicate induction and blue lines with arrow indicate activation of phytohormone signaling.

In the absence of brassinosteroid, the BRI1 receptor remains inactivated by several mechanisms (Figure 8). The cytoplasmatic C-terminal tail of BRI1 auto-inhibits its kinase activity [422], and the kinase domain is dephosphorylated by protein phosphatase 2A (PP2A), targeting the BRI1 pool for degradation through a negative-feedback loop [423]. PP2A is composed of a subset of cytoplasmatic β’ regulatory subunits (β′η, β′γ, β′ζ, and β′θ) [424] and is activated by methylation via SBI (SUPRESSOR OF BRI1), a leucine carboxylmethyltransferase (LCMT), reducing receptor abundance and BR signaling strength [423]. Another important negative regulation is performed by the inhibitory protein BKI1 (BRI1 kinase inhibitor 1), which binds to BRI1 and inhibits its activity [425].

BRI1 can bind brassinosteroids at the extracellular island domain [426], inducing its homodimerization and kinase activity [421,427,428,429], releasing the effect of the C-terminal tail [430], and leading to the trans-phosphorylation of the BKI1 inhibitor [425]. BKI1 dissociates from the membrane and interacts with members from the 14-3-3 phosphopeptide-binding proteins (Figure 8), allowing brassinosteroid signaling to be activated [425,431,432]. BRI1 then associates with another LRR receptor kinase, BAK1 (BRI1-associated kinase 1) [433,434] (Figure 8), also known as SERK3 (somatic embryogenesis receptor kinase 3). BAK1/SERK3 works as a brassinosteroid co-receptor, forming heterodimers with BRI1 [435,436] in the presence of the hormone [437]. Brassinosteroid-induced BRI1-BAK1 dimerization results in the positioning of their intracellular kinase domains in the right structure for competing with BKI1 and allows receptors’ transphosphorylation at multiple sites [427,438], forming a fully activated receptor complex that activates downstream events [427,438] (Figure 8). Two other members of the BAK1/SERK family, SERK1 [439] and SERK4/BAK7/BKK1 [440], have also been reported to be involved in BR signaling. Once fully activated, BRI1 phosphorylates CDG1 (CONSTITUTIVE DIFFERENTIAL GROWTH 1) and BSKs (BR SIGNALING KINASEs) proteins [438,439,440,441,442], both belonging to the receptor-like cytoplasmic kinase (RLCK) superfamily. These proteins are located at the plasma membrane by amino-terminal myristoylation [406]. Once phosphorylated by BRI1, CDG and BSK1 kinases phosphorylate the phosphatase BSU1 (BRI1 suppressor 1) [443,444]. BSU1 was identified in a *bri1* suppressor screening [445]. BSU1 and its homologues, the BSU-likes (BSLs), belong to a small family of protein phosphatases known as PPKL (protein phosphatase with kelch-like domains) [445]. Once activated, they can dephosphorylate and inactivate GSK3-like kinase BIN2 (BRASSINOSTEROID-INSENSITIVE 2), a negative regulator of the brassinosteroid signaling [444,446] (Figure 8). Although *BSU1* was the first gene to be functionally characterized and named in the family, it is specific to the Brassicaceae family and shows remarkable sequence variation, even among closely related species [447]. *BSL* genes, on the other hand, are highly conserved in all land plants [447].

BIN2 is a constitutively active kinase that acts as a central regulator of the brassinosteroid signaling cascade [443,448] (Figure 8). In arabidopsis, BIN2 and two close homologues, BIL1 (BRZ-INSENSITIVE-LONG 1) and BIL2, act redundantly in BR signaling since the triple loss-of-function mutant *bin2-3 bil1 bil2* showed constitutive BR responses [448]. Once dephosphorylated by BSU and BSL, BIN2 becomes unstable, is degraded by a proteasome-mediated degradation [449], and releases the signal transduction cascade to its activated state (Figure 8). BIN2 activity is also regulated by interaction with the F-box E3 ligase KIB1 (KINK SUPPRESSED IN BZR1-1D), which, in the presence of brassinosteroids, will promote BIN2 ubiquitination and proteasomal degradation (Figure 8). KIB1 also blocks BIN2 access to its substrate, the BES1 (BRI1 EMS SUPPRESSOR 1)/BZR1 (BRASSINAZOLE-RESISTANT 1) transcriptional factors [450]. When the brassinosteroid levels are low or absent, the active BIN2 will hyperphosphorylate the two key transcriptional factors involved in the brassinosteroid response BES1 and BZR1 (Figure 8). Both genes have been isolated as gain-of-function dominant mutants and suppress weak *bri1* mutant [451,452]. These mutants accumulate dephosphorylated BES1 and BZR1 proteins in the nucleus independently of brassinosteroids and display constitutive BR responses [451,452,453]. In their phosphorylated forms, BES1 and BZR1 cannot bind to DNA [454] and are retained at the cytoplasm by interaction with at least five of the twelve arabidopsis 14-3-3 proteins (14-3-3λ, 14-3-3κ.14-3-3ε, 14-3-3ϕ, and 14-4-3ω) [455] and the eight rice 14-3-3 proteins (GF14a-h; growth factor 14a–h) [456] (Figure 8).

In the opposite scenario, when the brassinosteroid levels are high, and BIN2 is inhibited, the BES1/BZR1 proteins are accumulated in their dephosphorylated forms (Figure 8). The dephosphorylation of BES1/BZR1 proteins is dependent on PP2A phosphatases’ activity [457]. Interestingly, the PP2A regulatory subunits involved in BES1/BZR1 dephosphorylation (PP2A β′α and PP2A β′β) are different from the ones involved in BRI1 dephosphorylation and are located at the nucleus [457]. The dephosphorylated forms of BES1/BZR1 will move to the nucleus, where they can bind to cis-acting elements in their target promoters, regulating gene expression [458,459,460,461] (Figure 8). In the arabidopsis genome, there are four other genes related to *BES1* and *BZR1*, named *BEH1–4* (*BES1/BZR1 homologs 1–4*), which have also been shown to operate in brassinosteroid signaling [458]. The promoters of the brassinosteroid biosynthesis genes display an enrichment in BRRE elements, where BES1 and BZR1 can bind as homodimers and repress its transcription, regulating both brassinosteroid biosynthesis and growth responses [459]. This operates as a negative-feedback loop to stop brassinosteroid signaling by the inhibition of the hormone production. Working as heterodimers with other transcriptional factors from the basic helix-loop-helix (bHLH) family, BES1 and BZR1 can bind to the E-box elements to activate brassinosteroid-induced gene expression [458]. The analysis of BL-induced gene promoters indicated that the E-box elements are enriched in many brassinosteroid-induced gene promoters [462].

BIM1 (BES1-interacting Myc-like 1) and its two homologues, BIM2 and BIM3, are members of the bHLH sub-family that are involved in brassinosteroid signaling. Although *bim1*, *bim2*, and *bim3* single mutants do not display any visible phenotype, the *bim1 bim2 bim3* triple mutants show a brassinosteroid mutant phenotype: shorter hypocotyls in light and dark growth conditions and higher sensitivity to brassinazole (a brassinosteroid biosynthesis inhibitor) in hypocotyl elongation assays in the dark [458]. On the other hand, *BIM1* overexpression can partially rescue the *bri1* mutant and can directly bind to the promoter of BR-induced genes [458]. Another transcription factor involved in the brassinosteroid response is known as BEE (brassinosteroid enhanced expression). In arabidopsis, the *BEE* genes are members of a subfamily of the bHLH, with 16 genes [463]. However, among the tested genes, only *BEE1*, *BEE2*, and *BEE3* are responsive to brassinosteroid [464]. Similar to several other components of the brassinosteroid signaling that do not result in steroid-deficient mutant phenotypes (probably for gene duplication and functional redundancy), *bee1*, *bee2*, and *bee3* single or double mutants do not display any visible phenotype. Only *bee1 bee2 bee3* triple mutant has a reduced response to brassinosteroid and was shown to be shorter and display a light-grown phenotype similar to the weak brassinosteroid-response mutant but not the dwarf phenotype. This suggests that although *BEE1*, *BEE2*, and *BEE2* are positive regulators, they are not absolutely required for brassinosteroid signaling [464].

Brassinosteroid signaling involves the critical step of signal attenuation. Excessive levels of BRI1 result in overly amplified brassinosteroid responses. To regulate this, plant U-box proteins PUB12 and PUB13 mediate BRI1 ubiquitination, triggering its internalization and subsequent degradation via the proteasome [465,466]. Signaling attenuation has been demonstrated to be an important step after brassinosteroid signaling. Since great amounts of BRI1 produce over-enhanced brassinosteroid responses, BRI1 internalization and degradation mediated by BRI1 ubiquitination by plant U-box proteins PUB12 and PUB13 have been shown to be an important step [465,466].

### 9.3. Brassinosteroids and ROS on Drought Response

Recent studies highlighted the importance of brassinosteroid signaling in drought adaptation [467]. In chickpea (*Cicer arietinum*), brassinosteroid treatment under water stress increased fresh and dry weight, the number of tillers, stem thickness, and root development [468]. In radish (*Raphanus sativus*) seedlings, it activated antioxidant enzymes, reducing drought stress effects [467]. In other species, such as sorghum (*Sorghum bicolor*), maize, and tomato, brassinosteroids enhanced chlorophyll accumulation, stomatal conductance, photosynthesis, and membrane stability, contributing to improved growth and yield [467].

In *B. distachyon*, *BRI1*-RNAi transgenic lines showed increased drought tolerance and increased expression of drought-induced genes [418]. The *bri1* loss-of-function mutation led to drought resistance in arabidopsis [469,470]. Similar results have been observed in tomato, where a mutant in the *SlBRI1* gene with weak brassinosteroid signaling (*abs*) exhibited drought tolerance. In comparison with control plants, *abs* mutants show a weak degree of wilting, higher water content in soil, and lower electrolyte leakage and MDA content. On the other hand, the overexpression of tomato *SlBRI1* reduces stomatal aperture, antioxidant enzyme activities, and the expression of stress-related genes, negatively modulating drought tolerance [471]. In maize, under drought conditions, *BRI1* RNAi-silenced transgenic lines display less wilting, higher survival rates, higher water content and water loss rate, higher stomatal closure, lower electrolyte leakage and MDA content, and a reduced photosynthetic decline rate in comparison with control plants. Opposite phenotypes have been observed in transgenic lines over-expressing *ZmBRI1*, indicating that *ZmBR1* negatively modulates drought tolerance in maize [472]. These observations indicate that water stress regulates brassinosteroid signaling through *BRI1* and that higher levels of the BRI1 receptor result in reduction in drought tolerance [470,471,472].

Overexpression of the *BRL3* gene, another member of the brassinosteroid receptor family, enriched in the vascular system, confers tolerance to drought stress. Although loss-of-function mutations in the BRI1 receptor also lead to drought resistance, this occurs at the expense of plant growth. However, overexpression of the BRL3 receptor confers drought tolerance without penalizing overall growth. Systematic analyses have revealed that, under drought stress, increased *BRL3* expression triggers the accumulation of osmoprotective metabolites, including proline and sugars [470]. Under control conditions, plants overexpressing the BRL3 receptor already exhibit a metabolic signature enriched in the accumulation of proline and sugars, which are classically correlated with stress tolerance. Under stress conditions, plants overexpressing *BRL3* also accumulate high levels of proline, GABA, tyrosine, trehalose, myo-inositol, and raffinose in a manner correlated with stress exposure. All these metabolites have previously been associated with drought stress tolerance. High levels of the raffinose family oligosaccharides (RFOs) and myo-inositol, known to be involved in the protection of biological membranes and free radical scavenging, were also observed in the roots of plants overexpressing the BRL3 receptor. The results strongly suggest that *BRL3* overexpression promotes the “priming” phenomenon, protecting plants from damage associated with the drought stress process [470].

*OsGSK1*, the rice orthologue of BIN2, was also shown to be involved in drought response. *OsGSK1*-knockout plants demonstrated more tolerance to several stresses, including drought [473]. In wheat (*Triticum aestivum*), a gain-of-function mutation in the *TaGSK3* gene (*TaGSK3-3D*) confers drought tolerance. *TaGSK3* encodes a SHAGGY-like kinase protein homologous to the arabidopsis BIN2. The gene was mapped and identified by the analysis of an Indian dwarf wheat cultivar (Sphaerococcum 1, S1) that is insensitive to brassinosteroid, displays upregulation of brassinosteroid biosynthetic genes, and enhances drouth tolerance [12]. *TaGSK3-3D* plants maintain higher relative water content through stomatal-regulated water loss and reduced ROS levels. Although *TaGSK3-3D* plants exhibited growth penalties under normal conditions, upon stress treatment, they displayed higher survival rates, plant height, and biomass compared to wild-type plants [474]. BIN2 and its homologues limit plant growth by inhibiting BES1/BZR1 activity; however, they enhance drought tolerance by phosphorylating SnRK2s, intensifying ABA signaling, and regulating other transcription factors involved in the activation of drought tolerance [475,476].

Under normal conditions, BES1 and the transcription factors WRKY46, WRKY54, and WRKY70 interact to promote the expression of genes related to growth and, at the same time, repress genes regulated by drought. On the other hand, drought stress conditions increase BES1 degradation, reducing its impact in plant growth and increasing drought response. In this condition, BIN2 phosphorylates WRKY54, allowing the expression of drought-inducible genes and reducing the growth stimulated by brassinosteroids [430]. BES1 also operates with the transcription factors RD26 (RESPONSIVE TO DESICCATION 26) [469] and TINY [477], coordinating plant growth and drought tolerance trade-off. RD26 and TINY are induced by drought and induce drought-responsive genes, increasing drought tolerance and inhibiting plant growth by inhibiting BES1. RD26 and TINY physically interact with BES1, inhibiting its transcriptional activity on brassinosteroid-regulated genes. On the other hand, BES1 represses the transcription of RD26, inhibiting drought responses [469,477]. BIN2 phosphorylates and stabilizes TYNY, performing a positive regulation in drought stress response [477].

In arabidopsis, a null mutant of the *BEH3* gene (*pca41*, *proline content alterative 41*) shows a drought-insensitive phenotype, higher levels of proline accumulation, and reduced levels of reactive oxygen species. Under osmotic stress, the mutant displays an increase in APX, CAT, and POD activities. Overexpression of *BEH3* results in an osmotic stress-sensitive phenotype that can be reverted by brassinolide application. BEH3 seems to operate together with the E3 ligase RZF1, and their expression mediates the water deficit response through brassinosteroid signaling and ubiquitination action [478]. Overexpression of the soybean BEH orthologue (*GmBEH3L1*) in arabidopsis also generates an osmotic-stress sensitive phenotype [479].

Interestingly, brassinosteroids signaling was also demonstrated as positively regulating drought stress. In wheat, the overexpression of *TaBZR2* results in drought-tolerant plants, while downregulation of the gene by RNAi generates plants that exhibit elevated drought sensitivity. Under drought conditions, *TaBZR2* RNAi plants display increased superoxide content, and it was demonstrated that *TaBZR2* confers drought tolerance by activating *TaGST1* expression [480]. Recently, a new gene involved in brassinosteroid signaling was identified through a genetic screening for a brassinazole-insensitive phenotype. Overexpression of *BIL9* (BRZ-INSENSITIVE LONG HYPOCOTYL 9) leads to drought tolerance, while *bil9*-knockout plants do not present any visible phenotype under drought conditions. *BIL9* overexpression induces the expression of ABA-induced and drought-responsive genes. BIL9 physically interacts with HOMEODOMAIN GLABROUS 11/ENHANCED DROUGHT TOLERANCE 1 (HDG11/EDT1), a transcription factor that promotes drought tolerance. BIL9/HDG11 interaction positively regulates BR-induced plant growth, promoting drought stress resistance [481].

Brassinosteroid signaling is also mediated by ROS. Brassinosteroid-induced hydrogen peroxide accumulation has been reported to be important for heat and oxidative stresses [482,483], stomatal movement [100,484], and salt tolerance [485]. Indeed, several components of the brassinosteroid signaling have been shown to be targets for ROS. The brassinosteroid co-receptor BAK1 was shown to be modified by glutaredoxin GRXC2, resulting in the inhibition of its kinase activity [486]. The kinase activity of BIN2 was also reported to be inhibited by nitric oxide [40]. Hydrogen peroxide leads to oxidative modifications in specific cysteine residues of BES1/BZR1, enhancing its transcriptional activity and promoting its interactions with other regulators of auxin and light signaling, such as ARF6 and PIF4. Oxidized BES1/BZR1 can be reduced by the thioredoxin TRXh5 [487]. Under hydrogen peroxide-deficient conditions, the number of genes regulated by BZR1 is greatly reduced, and the re-application of hydrogen peroxide partially restores the expression of the BZR1-regulated genes. Interestingly, BZR1 seems to repress ROS-related genes, contributing to feedback regulation and redox homeostasis [487]. Drought stress induces hydrogen peroxide production by brassinosteroid-activated NAPDPH oxidase, which may also activate BES1/BZR1 to regulate drought response [488].

Brassinosteroids signaling plays a pivotal role in balancing the trade-off between plant growth and drought tolerance. These hormones promote cell expansion and developmental processes under optimal conditions, enhancing overall growth. However, under drought stress, brassinosteroids trigger a complex adaptive response that prioritizes survival overgrowth. By modulating key signaling pathway, including those mediated by other phytohormones, brassinosteroids help plants adjust their metabolism, boost antioxidant defenses, and stabilize cellular structures, thereby minimizing the damage caused by water deficiency. As such, brassinosteroids are essential regulators that fine-tune the plant’s ability to conserve resources, optimizing both growth and stress resilience in challenging environments.

## 10. Strigolactones

Strigolactones are a small class of carotenoid-derived compounds that play a crucial role in suppressing shoot branching by inhibiting the outgrowth of axillary buds [489,490]. Initially, they were characterized as rhizosphere signals, helping root-parasitic plants detect and colonize their hosts [491]. However, strigolactones are now known to be involved in many other aspects of plant development [492]. They influence internode length [493,494], leaf morphology [495,496], leaf senescence [494,497], shoot gravitropism [498], stem thickness [499], as well as seed germination and early seedling development [500,501].

In the root system, strigolactones enhance the growth of primary roots, elongate root hairs, and promote the growth of rice crown roots [502,503,504]. Conversely, they inhibit adventitious root formation in eudicots [505,506,507]. Additionally, strigolactones play crucial roles in plant adaptive responses to environmental factors such as phosphate, nitrogen, light, drought, and high salinity [506,508,509,510,511,512].

### 10.1. Strigolactones Metabolism

In arabidopsis, *MORE AXILLARY GROWTH* genes *MAX1*, *MAX3*, and *MAX4* encode enzymes involved in the strigolactone-biosynthetic pathway. *MAX1* encodes a cytochrome P450 monooxygenase that is believed to be involved in a catalytic step downstream of *MAX3* and *MAX4* [509]. *MAX3* and *MAX4* encode carotenoid cleavage dioxygenase 7 (CCD7, EC 1.13.11.68) and CCD8 (EC 1.13.11.68), respectively. These enzymes catalyze sequential carotenoid cleavage reactions to produce an apocarotenone called carlactone, proposed as strigolactone precursor [513]. Indeed, an arabidopsis mutant in *MAX3* and *MAX4* exhibited a 70–75% reduction in strigolactones content [489]. After biosynthesis, a portion of strigolactones is exuded from the roots and enters the rhizosphere. In petunia, the PLEIOTROPIC DRUG RESISTANCE 1 (PDR1) protein played a crucial role in regulating the development of arbuscular mycorrhiza fungi and axillary branches [514]. PDR1 belongs to the ABC transporter family, which is also involved in the transport of other phytohormones, such as abscisic acid (ABA), auxin, and brassinosteroids [248,407,515,516]. Despite this, the mechanism underlying the transport of active strigolactones to the shoot remains unclear.

### 10.2. Strigolactones Signaling

**Figure 9:**
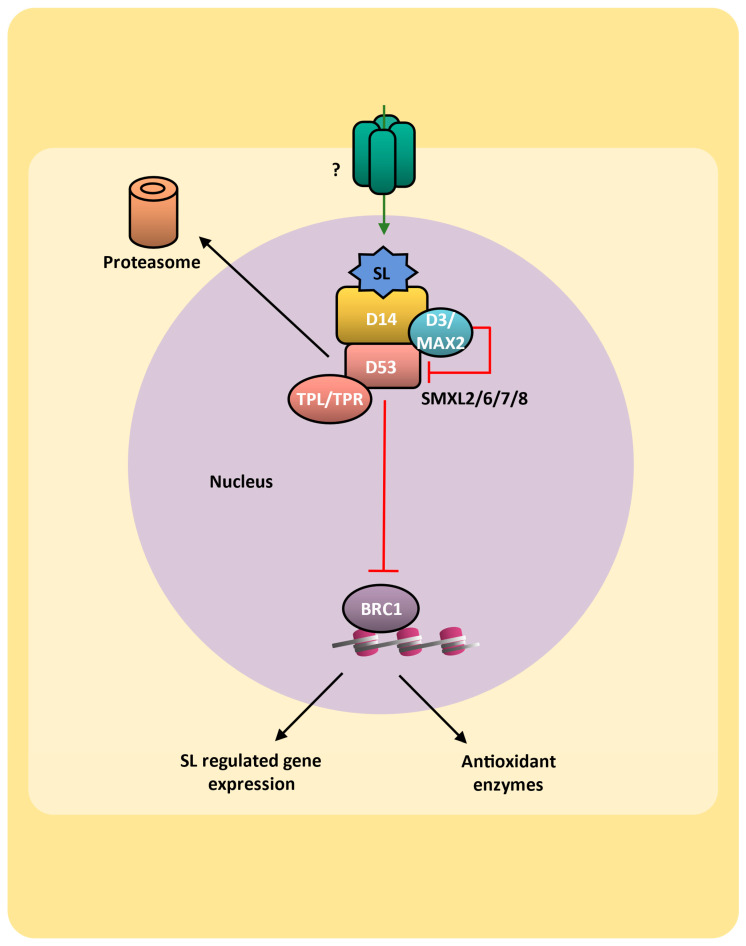
Strigolactone signaling. Strigolactones (SL) are perceived by the receptor D14, which is located in the nucleus. Although the mechanism of strigolactone influx into the cell is not yet fully elucidated, once inside, the hormone binds to D14, leading to its interaction with the repressor protein D53. This interaction facilitates the ubiquitination and proteasome-mediated degradation of D53, a process dependent on the F-box protein D3/MAX2. D3/MAX2, as an F-box protein, plays a crucial role in this degradation process, allowing the release of the transcription factor BRC1 from the D53-TPL/TPR corepressor complex. This release enables the activation of strigolactone-responsive genes. Strigolactones regulate key developmental processes, including the inhibition of shoot branching, modulation of root architecture, and promotion of symbiotic interactions with mycorrhizal fungi. The hormonal receptor is indicated in yellow, while activators of the signaling are shown in blue and inhibitors in salmon. Membrane channels/transporters are represented in green and transcription factors in purple.Red lines with bars represent inhibition of phytohormone signaling, black lines with arrow indicate induction, and green line with arrow indicates transmembrane transport. Proteins with identity still not confirmed are marked with “?”.

The perception of strigolactones requires a hormone-dependent interaction between the receptor D14 and the F-box proteins D3 (in rice) or MAX2 (in arabidopsis), which are F-box leucine-rich repeat proteins structurally similar to the auxin receptor TIR1 and part of the SCF ubiquitination complex. During this process, strigolactones are hydrolyzed into a covalently linked intermediate molecule (CLIM), triggering a conformational change in D14 that facilitates its interaction with D3/MAX2 [517] (Figure 9). This mechanism is distinct from that of all known active phytohormones, which are produced by biosynthesis enzymes and then reversibly bound by their receptors to initiate signal transduction.

Strigolactone signaling depends on the inhibitory proteins SMAX1 (suppressor of MAX2 1), SMXL6 (SMAX-like 6), SMXL7 (SMAX-like 7), and SMXL8 (SMAX-like 8). In the absence of strigolactones, SMAX/SMXL proteins interact with transcriptional corepressors TPL and TPRs, potentially repressing the activities of target transcription factors such as BRC1 (BRANCHED1) [518,519,520] (Figure 9). The SMAX/SMXL proteins share high similarity with rice D53 and are also referred to as D53-like SMXLs [518,521]. In the presence of strigolactone, D3/MAX2 acts as the substrate-recruiting subunit of the SCF-type ubiquitin–E3 ligase complex D14-SCFD3 [509], which leads to the degradation of the inhibitory protein D53 through the 26S proteasome system, thereby allowing the expression of genes related to strigolactone response, such as *BRC1*, which encodes a TCP-type transcription factor that acts downstream of MAX2 in the regulation of shoot branching [509].

Strigolactones also interacts with other hormonal signaling. It has also been demonstrated that strigolactones induce the expression of CYTOKININ OXIDASISE DEHYDROGENASE 9 (CKX9), promoting cytokinin degradation [522,523]; inhibit auxin transport [524]; and regulate PIN auxin efflux carriers [525]. In apple, strigolactones regulate shoot regeneration through interaction with other phytohormones [526]. Strigolactones treatment induces members of the auxin biosynthesis gene family *Yucca* as well as *Aux*/*IAA* and *ARF* transcriptional factors, suggesting that strigolactones increase auxin levels, inhibiting shoot regeneration. The hormonal treatment also inhibits the ABA receptor *PYL4* and the cytokinin oxidase GA2ox2 involved in the inhibition of active GA. Treatments with an inhibitor of strigolactones increase the expression of the brassinosteroid receptor *BRI1* [526]. These results suggest that, besides the reduction in cytokinin and increased auxin signaling, abscisic acid, gibberellin, and brassinosteroids might also be involved in the strigolactone-mediated inhibition of shoot regeneration [526].

### 10.3. Strigolactones and ROS on Drought Response

Strigolactones positively regulate drought and high-salinity responses in arabidopsis. Both strigolactone-deficient and strigolactone-responsive mutants exhibit hypersensitivity to drought and salt stress. Exogenous strigolactone treatment rescues the drought-sensitive phenotype of strigolactone-deficient mutants but not of strigolactone-responsive mutants. This treatment also enhances drought tolerance in WT plants, confirming the role of strigolactones as positive regulators in stress responses. Additionally, the *MAX3* and *MAX4* genes, which are involved in strigolactone biosynthesis, are significantly induced by dehydration in leaves [527].

Strigolactones promote the expression of stress-related genes *RD22*, *RD29a*, and *RD29b*, improving tolerance to drought [528]. In addition, strigolactones are also involved in fine-tuning the plant’s response to oxidative stress by enhancing the expression and activity of antioxidant enzymes such as SOD, CAT, and POD [529,530] and promoting the production of non-enzymatic antioxidants, including reduced ascorbate, glutathione, and phenolic compounds, which scavenge ROS and protect against oxidative damage [531,532]. This helps maintain a balanced cellular redox state and shields plants from oxidative stress induced by environmental factors [529]. Thus, during the stress response, strigolactones play a critical role in the balance between ROS detoxification and production, ensuring the activation of ROS-dependent signaling and protecting against oxidative stress. However, as the field of strigolactones research is still evolving, further studies are needed to elucidate the precise mechanisms of strigolactones action and their potential applications in agricultural systems.

## 11. Phytohormones Crosstalk During Drought Response

**Figure 10:**
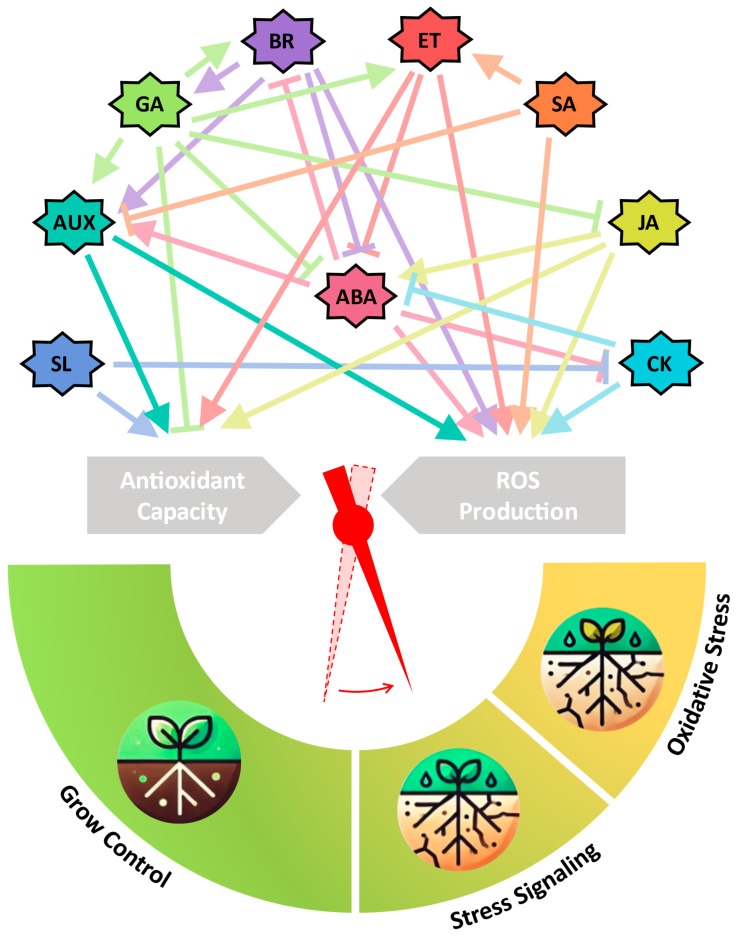
Hormonal crosstalk regulating ROS and antioxidant capacity. The crosstalk mechanisms among strigolactones (SL), auxin (AUX), gibberellin (GA), brassinosteroids (BR), abscisic acid (ABA), ethylene (ET), salicylic acid (SA), jasmonates (JA), and cytokinin (CK) signaling are very complex and may vary across species. Lines with arrows indicate activation, whereas lines with bars represent inhibition. Arrows and lines colors have been used according to the hormone color in the figure. These crosstalk mechanisms play a key role in regulating ROS production and antioxidant capacity in plant cells. Under environmental stresses, ROS production is induced, triggering different signaling that mediate the trade-off between growth control and stress responses. In these conditions, the activation of antioxidant defenses is essential to balance the redox status and prevent oxidative damage. During drought stress, both phytohormones and ROS act as second messengers in several stress signaling, which is engaged to reduce water loss through transpiration and enhance water uptake by the roots, including stomatal closure, root growth, leaf senescence, and osmoprotectant synthesis.

Although ABA is recognized as the main phytohormone related to drought, the outcomes of response and tolerance to adverse conditions result from a complex interaction of multiple hormonal actions [533]. The stress response pathways exhibit intricate connections, often converging on common elements, which is referred to as “crosstalk”. The term crosstalk denotes situations where different signaling share one or more elements, resulting in shared outputs [534] (Figure 10). The plants’ physiological responses to stressful environments, involving changes in biological processes, arise from antagonistic or synergistic interactions between various phytohormones [535]. Consequently, the development of tolerance in plants against drought emerges as a complex phenomenon involving intricate interactions across various cellular, molecular, and metabolic dimensions [8].

The ABA regulation of stomatal closure under abiotic stress is directly related to ethylene signaling, which can act as either a positive or negative regulator [101]. This mechanism is dependent on ABA concentration. When ABA is not sufficient, drought stress leads to ethylene synthesis, which then induces the accumulation of ROS synergistically with ABA, inducing stomatal closure through the synthesis of NO and the activation of the SLACs channel [98,536]. In this condition, ethylene is also able to induce ABA biosynthesis through ERF transcription factors. In different species, the overexpression of *ERFs* results in increased ABA content, rapid stomal closure, and enhanced abiotic stress tolerance [101]. On the other hand, ABA inhibits *ERF1* gene expression through a negative-feedback mechanism [537]. When ABA levels are high, ethylene starts to have an oppositive effect, inhibiting ABA-induced stomatal closure [98]: both exogenous ethylene and the ethylene-overproducing mutant *eto1-1* show a reduced ABA-induced stomatal closure in drought-stressed arabidopsis [538]. It has been suggested that, under these conditions, ethylene might trigger the production of flavonoids, which could decrease NADPH oxidase activity. This would subsequently inhibit the synthesis of ROS induced by ABA, ultimately preventing ABA-induced stomatal closure [539,540]. Ethylene also regulates ROS metabolism by modulating antioxidant enzymes such as SOD, CAT, APX, and GR [541,542]. Similarly, the ethylene precursor ACC induces APX, CAT, SOD, and POX activities and reduces lipid peroxidation [543].

Despite the initial observation of water stress-induced ethylene synthesis [544], some studies have yielded varied and sometimes contradictory results, suggesting that drought stress either fails to increase or reduces ethylene production [545]. The ABA-induced transcription factor ABI4 mediates the transcriptional repression of *ACS4* and *ACS8*, resulting in inhibition of ethylene biosynthesis during elevated ABA levels [98]. This is supported by the increased ethylene production in maize and tomato mutants deficient in ABA synthesis [546,547]. The combined ethylene–ABA stimulus results in half-open stomata, with diminished closure compared to the effect of the individual ABA or ethylene stimulus [548]. In this context, it was demonstrated that the inhibition of ethylene biosynthesis and perception can increase stomata closure, enhancing drought tolerance. In maize, the silencing of ACS decreases ethylene biosynthesis and improves drought tolerance [549]. Similarly, plants with reduced ethylene sensitivity, such as arabidopsis and maize overexpressing *ARGOS* genes [74] and arabidopsis mutants *etr1-1* and *ein2-1* [550], showed reduced water loss through transpiration and increased drought tolerance.

Despite this, reduced stomatal conductance and transpiration are not directly associated with drought tolerance, and the limitation of stomatal conductance is recognized as the primary factor contributing to the reduction in photosynthesis and oxidative stress during drought response [551,552,553]. Although reduced stomatal conductance leads to lower transpiration and water loss, it also restricts the supply of CO_2_ for RubisCO (EC 4.1.1.39) carboxylase activity, thereby limiting photosynthesis and diverting ribulose bisphosphate (RuBP) to photorespiration, which is the primary source of ROS in photosynthetic tissues [551,552,553]. Therefore, the regulation of ABA-induced stomatal closure, along with the activation of the antioxidant system through the ethylene signaling, plays a central role in ensuring an appropriate response to drought (Figure 10).

Under moderate drought conditions, ABA can positively regulate root system architecture, increasing primary root length and root hair density through its induction of auxin biosynthesis and transport in the primary roots [554,555,556,557]. Indeed, the knockout of the ABA catabolic gene *ABA8ox* in rice [556] or the exogenous ABA application in arabidopsis [555] increases the auxin flux towards the root, resulting in increased root length and drought tolerance. Low concentrations of ABA also facilitate primary root growth by mitigating PP2C-D-mediated inhibition of the apoplastic efflux of H^+^ through the AHA2 channel [558]. For effective hydrotropism, the ABA-induced kinase SnRK2.2 is required at the cortical cells in the root elongation zone, where it promotes cellular elongation, allowing differential growth [559]. On the other hand, at elevated concentrations, ABA inhibits root growth by disrupting auxin signaling in roots, repressing genes encoding transporters responsible for auxin translocation [560]. Taken together, these observations show that both auxin and ABA play crucial roles in shaping the root system architecture in response to water availability. Mild soil drying and low ABA concentrations stimulate root growth by inducing auxin signaling. However, as drought conditions intensify, and ABA and ROS levels increase, auxin signaling and root growth are suppressed. While this inhibition does not enhance water absorption, it serves as an adaptive strategy during severe drought, allowing plants to allocate resources toward preserving photosynthetic tissues, thereby maximizing their chances of survival [561].

ABA and brassinosteroids are known to act antagonistically, balancing growth and stress response. BIN2 kinase seems to be the central hub of this interaction since its activity is repressed by dephosphorylation mediated by PP2Cs, a part of the central core of ABA signaling [476,562]. Under drought conditions, the increase in ABA leads to the repression of the PP2Cs ABI1 and ABI2. This allows BIN2 to be an active kinase, attenuating growth responses due to the phosphorylation of BES1/BZR1 transcriptional factors, which reduces the transcription of the brassinosteroid-induced genes related to growth [476]. On the other hand, BIN2 also phosphorylates SnRK2s, intensifying ABA signaling [475,563]. ABA also negatively regulates the expression of the transcriptional factors BEE1, BEE2, and BEE3 [464].

Pioneer analyses of auxin and brassinosteroid co-regulation have demonstrated that the signaling of both hormones converges at the promoters of shared target genes [462,564]. BES1 and BZR1 also integrate brassinosteroid signaling with other phytohormone signaling, co-regulating plant growth and stress tolerance [565,566,567,568]. Promoter regions from several transcriptional factor genes involved in light, auxin, and gibberellin signaling are targets of BZR1 [460]. BES1 and BZR1 also interact with PIF and DELLA transcriptional factors, coregulating several genes and modulating cell elongation and photomorphogenesis [456,569,570,571].

The presence of jasmonic acid in the roots has been suggested as essential during water stress to increase ABA levels [27,572]. Despite this, jasmonic acid inhibits root growth and reduces meristem activity [573]. This inhibition appears to be mediated by the interaction between jasmonic acid and auxin signaling [574]. Upon jasmonic acid induction, MYC2 binds to the promoters of the auxin-responsive gene *PLT* (PLETHORA), responsible for stem cell niche maintenance and cell division, leading to suppression of its expression and thereby inhibiting root meristem activity [574]. On the other hand, jasmonic acid inhibition of root growth is negatively regulated by brassinosteroids [575], which act antagonistically to jasmonic acid during plant defense [576]. The antagonistic regulation of metabolic genes is the main feature of gibberellin and ABA interactions [574]. ABA-deficient mutants exhibit elevated gibberellin levels, highlighting ABA’s role in suppressing gibberellin metabolic genes (*GA3ox1/2* and *GA3ox1/2/3*) during seed germination. Conversely, gibberellin-deficient mutants show increased ABA levels by upregulating ABA biosynthetic genes (*ABA1*, *NCED6*, and *NCED9*) while downregulating the ABA catabolic gene (*CYP707A2*) [577,578]. Under water-limited conditions, gibberellin can inhibit ABA biosynthesis and stomatal closure [579]. Gibberellin is also recognized to antagonize ABA regulation of different developmental stages, including seed dormancy, seed germination, root growth, leaf development, flowering time, and responses to environmental cues such as light, temperature, and other abiotic stresses [580].

The gibberellin signaling appears to impair stress responses. The DELLA protein family, which inhibits gibberellin signaling in its absence, also promotes an ABA response by promoting the expression of the ABA transporter gene *ABA-IMPORTING TRANSPORTER 1.1* (*AIT1.1*) in guard cells [540] and activating ABI transcription factors [581]. Transgenic tomato plants overexpressing the constitutively active DELLA protein proceraΔ17 (proΔ17) showed decreased stomatal aperture and plant transpiration; however, these effects are suppressed in the ABA-deficient *sitiens* (*sit*) mutant, suggesting DELLA’s role is ABA-dependent [241]. Similarly, DELLA can bind and inhibit JAZ, the central negative regulator of jasmonic acid signaling [582]. Gibberellin-mediated degradation of DELLA contributes to improve JAZ inhibition of jasmonate-responsive gene expression [583,584] and jasmonate-mediated plant immune responses [585].

Despite antagonizing the stress-induced ABA and jasmonate responses, gibberellin acts synergically to auxin, brassinosteroids, and ethylene, exerting a pivotal role in the modulation of intricated molecular and cellular processes that allow plants to respond to environmental conditions [239]. Gibberellin can regulate the auxin transport by inducing the expression of the PIN-FORMED auxin transporters *PIN1*, *PIN2*, and *PIN3*. This mechanism has an important role in gravitropism and xylogenesis [294,586]. Indeed, arabidopsis gibberellin biosynthesis- and signaling-deficient mutants show reduced activity of PIN, and the wild-type phenotype is restored upon exogenous gibberellin application [587]. Additionally, DELLA protein RGA interacts with and inhibits the auxin-responsible factors ARF6, ARF7, and ARF8. Gibberellin enhances the transcription of auxin response by promoting DELLA degradation [568]. Similar mechanisms appear to allow gibberellin to positively regulate the transcription of brassinosteroids and ethylene-responsive genes. DELLA also regulates BES1 and BZR1 1transcription factors, which control the responses and are activated by gibberellin via DELLA degradation [588], indicating an important intersection between gibberellin and brassinosteroids signaling. In ethylene signaling, the degradation of DELLA mediated by gibberellin is accompanied by the inhibition of the stress-responsive genes *AP2/ERF* [239]. Additionally, gibberellin induces ethylene biosynthesis by inducing *ACS5* and *ACS8* [589].

Stress-induced ABA accumulation downregulates cytokinin biosynthesis through the MYB2 transcription factor, which relieves the repression of cytokinin signaling and activates ABA- and stress-inducible genes [590]. Indeed, ABA and cytokinins regulate bud outgrowth antagonistically [591]. Under drought conditions, tZ transport is impaired [592]. Key kinases in the ABA signaling, such as SnRK2.2, SnRK2.3, and SnRK2.6, directly phosphorylate several Ser residues of ARR5, a type-A ARR and inhibitor of cytokinin signaling. This phosphorylation stabilizes the ARR5 protein, enhancing drought tolerance by suppressing cytokinin signaling and by positively regulating ABA signaling in an SnRK2-dependent manner [593]. Despite acting antagonistically, during biotic stress response, cytokinins induce ROS production and stomata closure. This mechanism is not affected in the ost1-3 mutant, indicating that it occurs in an ABA-independent manner [160]. In arabidopsis, both ABA and drought downregulate the expression of *ARR1*, *ARR10*, and *ARR12* [397]. Additionally, ABA-activated ABI4 binds the promoters of *ARR6*, *ARR7*, and *ARR15*, further impairing their expression [594,595,596]. Thus, the ABA-mediated inhibition of cytokinin signaling can reshape the plant body by downregulating shoot growth while accelerating root growth. This allows the plant to reduce water loss and increase water uptake from deeper soil layers [590].

Conversely, elevated endogenous cytokinins suppress ABA signaling, thereby influencing a trade-off between growth and defense mechanisms. This reduced sensitivity to ABA under high cytokinins levels is thought to be mediated through type-B ARRs such as ARR1, ARR11, and ARR12, which interact directly with SnRK2s, inhibiting the kinase activity of SnRK2.6 [593]. Indeed, *AHK2*, *AHK3*, and *AHK4* defective mutants as well as type-B ARRs *ARR1*, *ARR10*, and *ARR16* exhibit higher sensitivity to ABA and display increased resistance to drought [397,597,598]. On the other hand, the type-A ARRs ARR4, ARR5, and ARR6 downregulate ABI5 expression, contributing to ABA response [595].

Strigolactones act as positive regulators of stress signaling networks and various ABA signaling by regulating the expression of many stress- and ABA-responsive genes involved in plant development and abiotic stress responses. Impaired strigolactone signal transduction also leads to the downregulation of CKX genes, which are necessary for cytokinin degradation [527]. These findings suggest that coordinated crosstalk between strigolactones, ABA, and cytokinins signaling networks regulates plant adaptation to adverse environmental conditions. Consistent with the drought-sensitive phenotype, max mutants, which show disrupted strigolactone biosynthesis, displayed increased leaf stomatal density compared to wild-type plants and exhibited slower ABA-induced stomatal closure. Relative to the wild type, max mutants showed a higher rate of leaf water loss during dehydration and reduced ABA responsiveness during germination and post germination, underscoring the role of strigolactones as positive regulators of ABA signaling [527].

## 12. Concluding Remarks

In summary, plant hormones play a crucial and multifaceted role in mediating plant responses to drought stress. Unlike animal hormones, which often have a single, well-defined function, plant hormones operate across a spectrum of functions, with their effects depending on the interplay between different signaling. This complexity allows plants to finely tune their responses to the timing and intensity of drought stress.

ABA is recognized as the primary regulator of drought responses, driving stomatal closure through ROS production and optimizing root tissue patterning to minimize water loss and enhance uptake. Ethylene complements ABA at low concentrations but opposes its effects when ABA levels are high. Salicylic acid also supports ABA by inducing ROS-mediated stomatal closure when ABA signaling is insufficient; jasmonates improve ABA accumulation, aiding stomatal closure and root adaptations; and strigolactones enhance drought tolerance by amplifying ABA responses, regulating stress-related genes, and strengthening antioxidant defenses. In contrast, gibberellins and cytokinins counteract ABA by suppressing stomatal closure, ROS production, and growth under stress. Auxins and brassinosteroids act mainly in the balance between growth and stress adaptation, optimizing root architecture and enhancing antioxidant defenses (Figure 10).

These nine classes of plant hormones interact synergistically, coordinating a comprehensive response to stress while balancing growth and survival. These hormones do not function in isolation but rather through intricate networks where crosstalk between pathways is vital. Notably, ROS often mediate these signaling, acting as key molecular signals that integrate stress responses (Figure 10). These mechanisms are essential for plant stress responses and occur differently depending on the intensity of the stress. In moderate stress conditions, such as mild drought, the crosstalk between various signaling promotes coordinated responses that enable the plant to maintain growth and adapt to the environment, such as auxin activation to stimulate root growth (Figure 10). However, under severe stress, like intense drought, these crosstalk mechanisms shift, prioritizing the preservation of photosynthetic tissues and minimizing cellular damage. The trade-off between growth and stress response reflects the differential allocation of energy resources, allowing the plant to prioritize survival under stress without irreversibly compromising development (Figure 10). In this context, the interaction between different phytohormones and ROS signaling can inhibit processes such as root growth, favoring adaptive strategies that ensure the plant’s survival by redirecting resources to essential vital functions.

From an evolutionary perspective, the ability of plants to modulate their responses through such diverse and interconnected hormonal signals has been crucial for their adaptation to fluctuating environments. As we face increasing climatic extremes and the pressing need to expand agricultural production to meet growing food demands, understanding these hormonal pathways offers the potential to identify new biotechnological targets. This knowledge is essential for developing crops with enhanced resilience to drought, ultimately contributing to global food security and sustainable agricultural practices.
